# 3D Network exploration and visualisation for lifespan data

**DOI:** 10.1186/s12859-018-2393-x

**Published:** 2018-10-23

**Authors:** Rolf Hühne, Viktor Kessler, Axel Fürstberger, Silke Kühlwein, Matthias Platzer, Jürgen Sühnel, Ludwig Lausser, Hans A. Kestler

**Affiliations:** 1 Institute of Medical Systems Biology - Ulm University, Albert-Einstein-Allee 11, Ulm, 89081 Germany; 2 Leibniz Institute on Aging - Fritz Lipmann Institute, Beutenbergstr. 11, Jena, 07745 Germany; 3 Institute of Neural Information Processing - Ulm University, Albert-Einstein-Allee 11, Ulm, 89081 Germany

**Keywords:** Lifespan, Ageing, Gene network, 3D visualization, Ageing factor database, AgeFactDB, Differentially expressed genes

## Abstract

**Background:**

The Ageing Factor Database AgeFactDB contains a large number of lifespan observations for ageing-related factors like genes, chemical compounds, and other factors such as dietary restriction in different organisms. These data provide quantitative information on the effect of ageing factors from genetic interventions or manipulations of lifespan. Analysis strategies beyond common static database queries are highly desirable for the inspection of complex relationships between AgeFactDB data sets. 3D visualisation can be extremely valuable for advanced data exploration.

**Results:**

Different types of networks and visualisation strategies are proposed, ranging from basic networks of individual ageing factors for a single species to complex multi-species networks. The augmentation of lifespan observation networks by annotation nodes, like gene ontology terms, is shown to facilitate and speed up data analysis. We developed a new Javascript 3D network viewer JANet that provides the proposed visualisation strategies and has a customised interface for AgeFactDB data. It enables the analysis of gene lists in combination with AgeFactDB data and the interactive visualisation of the results.

**Conclusion:**

Interactive 3D network visualisation allows to supplement complex database queries by a visually guided exploration process. The JANet interface allows gaining deeper insights into lifespan data patterns not accessible by common database queries alone. These concepts can be utilised in many other research fields.

**Electronic supplementary material:**

The online version of this article (10.1186/s12859-018-2393-x) contains supplementary material, which is available to authorized users.

## Background

In ageing research, the lifespan of an organism is an indicator for determining factors that play a role in this process. These ageing factors (AFs) can be genes, chemical compounds or other factors like dietary restriction. Usually, they are examined under different experimental conditions in model organisms like the worm (*Caenorhabditis elegans*), yeast (*Saccharomyces cerevisiae*), fruit fly (*Drosophila melanogaster*), mouse (*Mus musculus*), and many others. The results of these experiments may be extracted from the scientific literature in the form of lifespan observations (LOs). They describe the effect of interventions at AFs on the lifespan of the model organism.

In a lifespan experiment, a single AF or a combination of two or more AFs can be involved. The intervention can be different for each AF. For example, a knock-out of gene *A* could be coupled to the overexpression of gene *B*. Also, AFs can be involved in different experiments together with different other AFs. For example, some genes like *daf-2* and *daf-16* from *C. elegans* were tested in several hundred AF combinations and various interventions, e.g. [1, 2]. The effects on the lifespan of the organism may also differ drastically.

### 3D Network visualisation

Dealing with this heterogeneity and the complexity of the relationships between the LOs is a major challenge in gaining a comprehensive overview or in generating an integrative model. Visualisation techniques can aid in analysing this complex data [3–5] and also help to generate new hypotheses not only on a quantitative level [6].

In line with these supportive approaches on sets, network visualisations can assist in organising vast amounts of data according to known relationships or properties. 3D visualisation can help researchers on special occasions in this task, although 2D representations should generally be preferred [7]. In the lifespan network visualisation context we present here, 3D networks outperform their 2D counterparts regarding compactness and layout. While 3D embeddings allow a compact representation of hundreds or thousands of nodes, 2D embeddings result in a significant expansion, increasing navigation costs (see Additional file 1: Figure S1). Furthermore, it is known that any finite graph can be embedded into a three-dimensional space such that no pair of edges crosses [8]. As LO networks cannot be guaranteed to be planar graphs, 2D embeddings might also result in intersecting edges, while in 3D these non-intersecting representations exist [8].

Additionally, psychophysical experiments provide evidence that the human primary visual processing system is specifically designed to process 3D information. Nakayma et al. indicate the parallel processing of attribute information like the colour from different depth planes [9]. Enns et al. give evidence that 3D objects with lightning-related depth cues accelerate the visual search in comparison to 2D objects [10]. Xu et al. report an increased capacity of the visual short-term memory (VSTM) [11] when objects are distributed between different layers [12].

The benefits of 3D network representations come at the risk of visual occlusion and possible perspective distortion [13]. Due to the layered structure of 3D representations, elements in the foreground can mask elements in the background. Which can be overcome by interactive graph manipulation such as zooming, rotating, panning and filtering.

Perspective distortions might occur when object sizes and distances are modified for both data visualisation and perspective depth effects. They can be omitted by ignoring depth calculations. Similar risks, such as diminished legibility of text, can be avoided by excluding these objects from other perspective transformations (i.e. rotations).

Overall 3D visualisation has many advantageous unique selling points. Its disadvantageous can be mitigated via interactive graph exploration and manipulation.

### AgeFactDB

The public JenAge Ageing Factor Database AgeFactDB [14] contains LOs for AFs. Table 1 provides an overview of the number of AFs and observations by type (lifespan, other ageing phenotypes, homology analysis) and by their ageing relevance evidence type (experimental, computational).

**Table 1:** AgeFactDB Statistics. Overview of the number of AFs and observations in AgeFactDB Release 1 by type and by their ageing relevance evidence type

	Ageing relevance evidence			
	Experimental	Computational	Both	Any
**Ageing factors**	**2743**	**14437**	**581**	**16599**
Genes	2594	14437	581	16450
Chemical compounds	91	-	-	91
Other factors	58	-	-	58
**Observations**	**8159**	**1452**	**-**	**9611**
Lifespan (structured)	7219	-	-	7219
Ageing phenotype (unstructured text)	940	-	-	940
Homology analysis	-	1452	-	1452

Computational evidence refers to homology analysis observations, each resembling a homology group from the HomoloGene database [15]. The boldfaced numbers are the sums of the follow up rows

Currently, the core of AgeFactDB is a collection of about 2600 genes for which LOs and other experimental evidence were gathered from experiments with different model organisms (experimental AFs). This set was extended by about 14,000 genes gained in a homology analysis using data from the homology database HomoloGene [15] (putative AFs).

Overall, AgeFactDB contains about 9500 observations. About 1000 are free-text descriptions of ageing phenotypes, and about 7000 are structured LOs. Besides, there are about 1500 homology analysis observations, each resembling a homology group.

As an example for structured LOs, Table 2 shows observation OB_000094 [16] involving the genes *FOB1*, *SIR2* and *TOR1* from *S. cerevisiae*. The deletion of these three genes resulted in a 33.5% increase in lifespan. Note that there are two different types of lifespan defined for yeast: chronological lifespan and replicative lifespan [17]. The chronological lifespan is the number of days which a specific yeast cell is living. The replicative lifespan is a measure of the total number of daughter cells generated by a mother cell [18].

**Table 2:** Example Observations from AgeFactDB

**Free-text Ageing Phenotype Observation (Data Type 1) – OB_006092**	
**Species**	*Mus musculus*
**Gene Symbol**	Trp63
**NCBI Gene ID**	22061
**Other Ageing Factor Name**	-
**Description**	Heterozygous mice have a shortened lifespan and display features of accelerated ageing.
**PubMed**	16107615
**Source**	GenAge
**Observation Stable ID**	OB_006092
**Structured LO (Data Type 2) – OB_000094**	
**Species**	*Saccharomyces cerevisiae*
**Strain**	BY4742
**Gene**	FOB1 (NCBI Gene ID 851688) - allele type: deletion/null;
	SIR2 (NCBI Gene ID 851520) - allele type: deletion/null;
	TOR1 (NCBI Gene ID 853529) - allele type: deletion/null;
**Compound**	-
**Other Ageing Factor**	-
**Significant Lifespan Effect**	increased
**Change**	33.47%
**Observed Lifespan**	32.7
**Reference Lifespan**	24.5
**Lifespan Unit**	divisions
**Measure**	mean
**Temperature**	30
**Temperature Unit**	°C
**Sex/Mating Type**	matalpha
**Description**	Deletion of TOR or SCH9 increase life span of sir2 fob1 double mutant cells. SIR2 and FOB1 are believed to act in a single genetic pathway to promote replicative life span by reducing the accumulation of extrachromosomal rDNA circles in the mother cell [PubMed ID 10521401]. Since dietary restriction also increases the life span of sir2 fob1 double mutant cells [PubMed ID 15328540], this supports the model that TOR1, SCH9, and dietary restriction act in a single pathway that is distinct from SIR2, FOB1, and extrachromosomal rDNA circles.
**Source**	Lifespan Observations Database (ID: 1093)
**Observation Stable ID**	OB_000094
**Homology Analysis Observation – OB_008235**	
**Number of Experimentally Confirmed Ageing-related Genes in Homology Group**	1
**Description**	The HomoloGene homology group 38185 contains 1 gene with experimental evidence for ageing relevance (C48E7.2 - Caenorhabditis elegans) and 12 other genes.
**Homologs**	POU1F1 *Gallus gallus* (NCBI Gene ID 374215);
	POU1F1 *Canis lupus familiaris* (NCBI Gene ID 403753);
	POU1F1 *Pan troglodytes* (NCBI Gene ID 470861);
	POU1F1 *Bos taurus* (NCBI Gene ID 282315);
	POU1F1 *Macaca mulatta* (NCBI Gene ID 719349);
	Pou1f1 *Mus musculus* (NCBI Gene ID 18736);
	Pou1f1 *Rattus norvegicus* (NCBI Gene ID 25517);
	pou1f1 *Danio rerio* (NCBI Gene ID 405777);
	POU1F1 *Homo sapiens* (NCBI Gene ID 5449)
**Observation Stable ID**	OB_008235
**Source**	AgeFactDB Homology Analysis
**Homology Source**	HomoloGene homology group 38185

### 3D Network viewer

Aside from commercial tools, there are a few other freely available 3D visualisation tools. NetworkX [19] and iGraph [20] are examples for software packages that offer 3D network layout algorithms and the generation of static network images. The Javascript library vis.js provides its rudimentary viewer which has a user interface that enables only rotation, zoom, and translation of the network model [21]. Vanted [22] and SeeNet3D [23] are examples of specialised viewers for the domains of metabolic pathways and communication. The Cy3D plugin [24] for Cytoscape [25], a 2D network viewer in the biomedical domain, provides a 3D rendering engine. It is only suitable for small networks since it only supports rotation and zoom and no translation. BioLayout Express 3D [26] provides a functional layout algorithm for more extensive networks, the Fast Multipole Multilevel Method (FMMM) algorithm [27]. Nonetheless, the development of BioLayout Express 3D as a freely available tool ceased several years ago.

In the following, we present different types of networks and visualisation strategies for LO data. We show the benefit of augmentation of AF/LO networks by annotation nodes compared to AF annotation. Annotation nodes can be for example gene ontology (GO) [28] term nodes and KEGG pathway [29] nodes.

We also present a new Javascript network viewer with a customised interface for the visualisation of lifespan data from AgeFactDB, combined with user-provided genes of interest, for example, a list of genes differentially expressed during ageing. By two concrete example applications, we demonstrate the usefulness of the visualisation strategies and the network viewer.

## Methods

### JANet

JANet (Javascript AgeFactDB Network-viewer) is a specialised Javascript 3D network viewer for the visualisation of ageing-related network data from AgeFactDB. JANet extends the original design of the AgeFactDB by an interactive component allowing the user to browse the content of the database in a 3D graph representation. It facilitates the navigation through the data corpus of experimental evidence, citations and other background information via natural 3D movements and a well organised set of graph manipulations. JANet is also an interface allowing an untrained researcher to relate his data with the data corpus of AgeFactDB. By incorporating gene lists of interest in the original networks, JANet provides an embedding in the domain of ageing research.

**Fig. 1:**
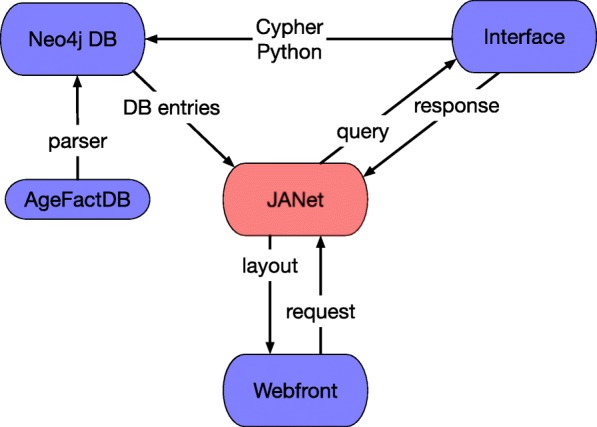
JANet Components. JANet consists of three major parts: The graph database Neo4j, the query interface and the web frontend. A parser processes the AgeFactDB database and converts its content in Neo4j graphs. The interface uses Python and the graph query language Cypher to access and query the Neo4j database. Requests from the web frontend are submitted to JANet and responses visualised on the web pages

As frontend, JANet provides a responsive HTML/ Javascript web browser interface, for an overview see Fig. 1. For the visualisation, it utilises the 3D Force-Directed Graph web component, based on ThreeJS/WebGL [30, 31] for 3D rendering and d3-force-3d as the underlying physics engine for generating the network layout. As the backend it has Python scripts and a Neo4j graph database [32].

#### Interface

**Fig. 2:**
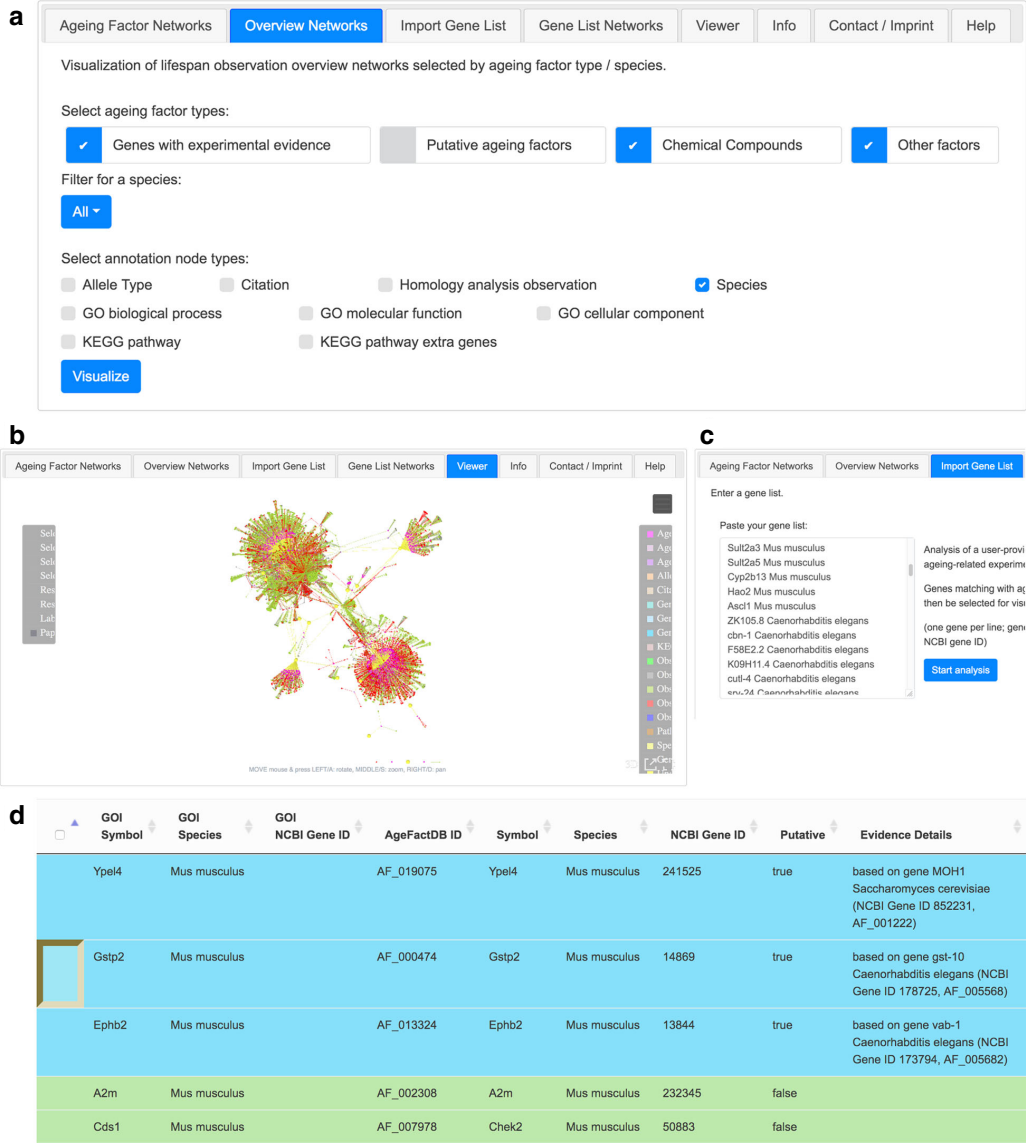
JANet Graphical User Interface. **a** Screenshot of the JANet graphical user interface. The tab *Overview Networks* is selected. **b** The *Viewer* tab of JANet. The network containing all ageing factors (AFs) having lifespan observations (LOs), all LOs, and the corresponding species is shown. **c** The *Import Gene List* tab allows the user to provide his own gene list of interest. Those genes that are linked to AFs in AgeFactDB are afterwards listed in the Gene List Network tab. **d** The *Gene List Network* tab contains a list of the genes of interest that are known AFs or homologous AFs. It can be used to generate user specific networks

The primary interface of JANet is structured into eight tabs which provide access to the main viewer for graph visualisation and the different options for network generation (Fig. 2a). Networks are generated according to three principles focusing on overviews of AFs, the inspection of individual AFs and the interaction of AFs with user-specified genes of interest. The interface additionally provides a statistical summary of the current database of AgeFactDB, help sides and the imprint. Generally, actions like node colouring and rendering work in the viewer on the currently selected nodes, enabling graph manipulations of node properties individually to create custom views.

#### Viewer

The *Viewer* tab provides the 3D graph representation of a chosen network and basic operations for graph editing (Fig. 2b). JANet comprises various options for customising the graph design and manipulating the graph layout. Among them, for example, changing the node colour and size. Network nodes can be marked with key information like the name or the lifespan change value. By hovering above a node with the mouse pointer, more detailed information about the node are given in the upper left corner of the viewer. The node itself is highlighted by a light blue halo. A white halo is shown when a node is selected. By selecting a region of interest (bounding box) sets of nodes can be selected or highlighted. The network can be restricted to the selected node and its direct neighbours. The network viewer provides alternative view options. It can be expanded to a fullscreen mode or send to an independent browser tab/window. In this way, several networks can be analysed simultaneously. For a better 3D impression the viewer also offers a stereo view option.

#### Overview networks

Rather holistic networks on all AFs of the AgeFactDB are generated in the tab *Overview networks*. The selection of AFs may be stratified according to the type of AF and the corresponding species. The tab also provides options for augmenting the graph with different kinds of annotation nodes. For example, they can provide additional information about the allele types or species. The constructed graph can afterwards be manipulated within the network viewer. Figure 2a gives an example of an overview network. It contains all AFs having LOs, all LOs, and the corresponding species.

#### Ageing factor networks

The networks generated by the tab *Ageing Factor Networks* are focused on one particular AF which is embedded either in its *direct* or *complete neighbourhood*. The functionality of this tab is comparable to the functionality of the tab for overview networks. Additionally, single AFs can be selected from a dropdown list.

#### Gene list selection / Network

JANet can be used as an interface for an integrative analysis combining the existing database of AgeFactDB and an user-specified list of genes of interest. Users can provide their list in the tab *Import Gene List* (Fig, 2c). A query to AgeFactDB returns a list of exact matches to ageing factors with experimental evidence and putative ageing factors. In the tab *Gene List Network* these results can be screened and edited (Fig. 2d). This tab also starts the network generation.

### Network layout algorithms

Force layout algorithms position the nodes of a graph in two-dimensional or three-dimensional space so that all edges are of more or less equal length and as few crossing edges as possible are produced. Repulsive or attractive forces are assigned to edges and nodes based on their relative position. By minimising their energy the layout is generated step wise. Within JANet we utilise three different algorithms.

The 3d-forced-layout (3dFL) algorithm module implements a velocity Verlet numerical integrator for simulating physical forces on nodes. It is a numerical method used to integrate Newton’s equations of motion [33]. In contrast the Fruchterman-Reingold (FR) algorithm focuses on even distribution of vertices, a minimal number of edge crossings and uniform edge length [34]. The fast multipole multilevel method (FMMM) is especially designed for separating substructures in large graphs [26].

### LO network visualisation techniques

We use LO Networks as basic visualisation technique for the integration of different lifespan experiments. In these networks LOs and AFs are represented by nodes. Edges between both types of nodes link the AFs with the LOs in which they were involved. Multiple AFs can be connected to one LO and vice versa.

In order to facilitate the visual navigation, we utilise a colour code for the different node types. In the special case of LO nodes the colour indicates the direction of the lifespan change. An increased, decreased or unchanged lifespan is indicated by green, red or grey colour. The node size can be proportional to the quantitative lifespan change or other quantitative measures like the number of connections (degree). The edge colour is usually inherited from the node of a pair whose colour carries specific information for the other node. For AF/LO edges this is the LO node, whose colour usually indicates the direction of the lifespan change.

**Fig. 3:**
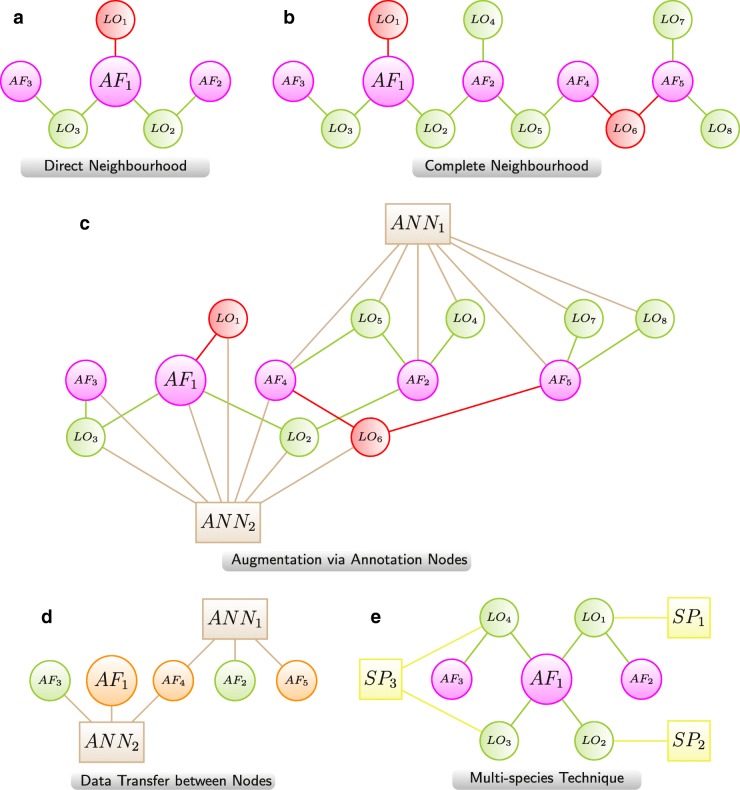
LO Network Visualisation Techniques. Techniques for the integrated visualisation of lifespan experiments as networks. LOs and AFs are represented by nodes. Node colour and size are used to speed-up the lookup of node properties by visual perception. **a** DirectNeighbourhood: For focusing on a specific AF only the direct neighbourhood of the AF is visualised. This includes the AF in the focus (*AF*_1_), all directly linked LOs (*LO*_1_,*LO*_2_,*LO*_3_), and all other AFs linked directly to these LOs (*AF*_2_,*AF*_3_). **b** Complete Neighbourhood: For a more comprehensive view the direct neighbourhood network is extended iteratively by including further AFs (*AF*_4_,*AF*_5_) that were observed together with the neighbours (*AF*_2_,*AF*_3_) of the AF in the focus (*AF*_1_) and their LOs (*LO*_4_,*LO*_5_,*LO*_6_,*LO*_7_,*LO*_8_). **c** Augmentation via Annotation Nodes: Additional information for AF and LO nodes is integrated by annotation nodes (*ANN*_1_, *ANN*_2_). They provide the information directly in a visual form and result in a reorganisation of the network layout. **d** Data Transfer between nodes: The data transfer between nodes can help to reduce the complexity of a network by removing nodes while retaining some of their data. Here all 8 LOs were removed after transforming the directions of the lifespan changes into a new colour scheme for the 5 AFs. **e** Multi-species: A special case of essential augmentation with species nodes for networks focusing on AFs that can be linked to multiple species, like chemical compounds. For a clearer view the AFs are linked only indirectly to the species nodes by the LOs: *AF*_1_ to *SP*_1_ via *LO*_1_, to *SP*_2_ via *LO*_2_, and to *SP*_3_ via *LO*_3_ and *LO*_4_;*AF*_2_ to *SP*_1_ via *LO*_1_;*AF*_3_ to *SP*_3_ via *LO*_4_. Color scheme (**a, b, c, e**):  AF,  LO - increased lifespan,  LO - decreased lifespan,  annotation,  species Color scheme (**d**):  annotation,  AF - linked only to LOs with increased lifespan,  AF - linked to LOs with highly mixed lifespan changes (increased and decreased >20%)

We present several visualisation techniques for LO networks (Fig. 3). In the “Results and discussion” section we present example networks, based on AgeFactDB data, to demonstrate the usage of these techniques.

#### Direct neighbourhood

For focusing on a specific AF only the direct neighbourhood of the AF is visualized (Fig. 3a). This includes the respective AF, all directly linked LOs, and all other AFs linked directly to these LOs.

This network type provides a compact view of the effects of all LOs in which an AF is involved directly.

In the network in Fig. 3a the AF in the focus is *AF*_1_. *AF*_1_ is linked to 3 LOs (*LO*_1_−*LO*_3_). *AF*_2_ is linked to *LO*_2_ because it was tested together with *AF*_1_ in the corresponding experiment. The same applies to *AF*_3_ and *LO*_3_. No further AFs were tested in any of the 3 LOs.

In *LO*_1_ the lifespan was decreased, indicated by the red node colour. In *LO*_2_ and *LO*_3_ the lifespan was increased, indicated by the green node colour.

#### Complete neighbourhood

The direct neighbourhood network is extended iteratively by including further AFs that were observed together with the neighbours of the AF in the focus (Fig. 3b). In this way the complete neighbourhood is included in the visualisation. The resulting network can be seen as the largest connected subgraph including the AF in the focus and all directly or indirectly connected LOs and AFs.

The complete neighbourhood network provides an overview on all experimentally analysed AF combinations and their effects on lifespan.

The direct neighbourhood network visualisation from Fig. 3a was expanded to the complete neighbourhood network visualisation in Fig. 3b. In the first expansion step *LO*_4_ and *LO*_5_ were added, linked to *AF*_2_ but not to *AF*_1_. Also *AF*_4_ was added, linked to *LO*_5_ together with *AF*_2_. In the second expansion step *LO*_6_ and *AF*_5_, linked also to *LO*_6_, were added. In the final expansion step *LO*_7_ and *LO*_8_ were added, both linked to *AF*_5_.

#### Augmentation via annotation nodes

LO networks are augmented by additional information on either the AFs or the LOs or both. This is achieved by additional annotation nodes (ANNs). They provide the additional information directly in a visual form for all nodes at once. The augmented network visualisation in Fig. 3c shows an annotated version of the complete neighbourhood visualisation from Fig. 3b. The annotation node *ANN*_1_ was linked to the 3 AFs (*AF*_2_,*AF*_4_,*AF*_5_) and to the 4 LOs (*LO*_4_, *LO*_5_, *LO*_7_, *LO*_8_). The annotation node *ANN*_2_ was linked to the 3 AFs (*AF*_1_,*AF*_3_,*AF*_4_) and to the 4 LOs (*LO*_1_−*LO*_3_, *LO*_6_). This resulted in two groups around the annotation nodes *ANN*_1_ and *ANN*_2_, connected by the nodes *AF*_4_, *LO*_2_, and *LO*_6_.

#### Data transfer between nodes

The data transfer between nodes is especially useful for reducing the complexity of a network by removing the nodes from which data was transferred. The transfer enables to retain some information from the removed nodes.

Figure 3d shows a reduced version of the network from Fig. 3c. The collected qualitative lifespan change information from all LOs connected to an AF was transformed into a new colour for the AF. *AF*_2_ and *AF*_3_ are linked only to green LO nodes (indicating an increased lifespan) and got green as new colour. *AF*_1_, *AF*_4_, and *AF*_5_ are linked to 2 or 3 green LO nodes and 1 red LO node (in the latter case indicating a decreased lifespan). These mixed effects were transformed into orange as new node colour.

The transferred data can also be used to define a new node size, which may be for example proportional to the maximal observed lifespan change. This information can already be helpful even if the LOs are not removed.

#### Multi-species technique

In contrast to genes, chemical compounds and other AFs can be linked to LOs of multiple species. This requires to augment such networks by species nodes (SP) that are linked to the corresponding LOs. We propose to leave out the links between the AFs and the species nodes. This will result in a much clearer and less complex network view, grouping the network clearly according to the involved organisms.

Because of the importance of the species information in these networks and the additional restriction to one type of species links we defined *Multi-species* as a separate technique.

The multi-species network visualisation in Fig. 3e contains *AF*_1_ as multi-species AF. It is linked indirectly to all 3 species nodes (*SP*_1_−*SP*_3_) by 4 LOs (*LO*_1_−*LO*_4_). In *LO*_1_ also *AF*_2_ is involved, and in *LO*_2_ also *AF*_3_ is involved.

The layout shows 3 small groups centred around the multi-species node *AF*_1_.

## Results and discussion

After the basic description of the visualisation techniques and network types given in the “Methods” section, we first present concrete examples for some of the visualisation techniques introduced in the “Methods” section followed by use cases how these techniques were applied with JANet to solve specific tasks.

### LO network visualisation examples

We show examples for the application to lifespan data for *S. cerevisiae* and *C. elegans*. All LOs were taken from AgeFactDB.

#### Example 1: direct neighbourhood

**Fig. 4:**
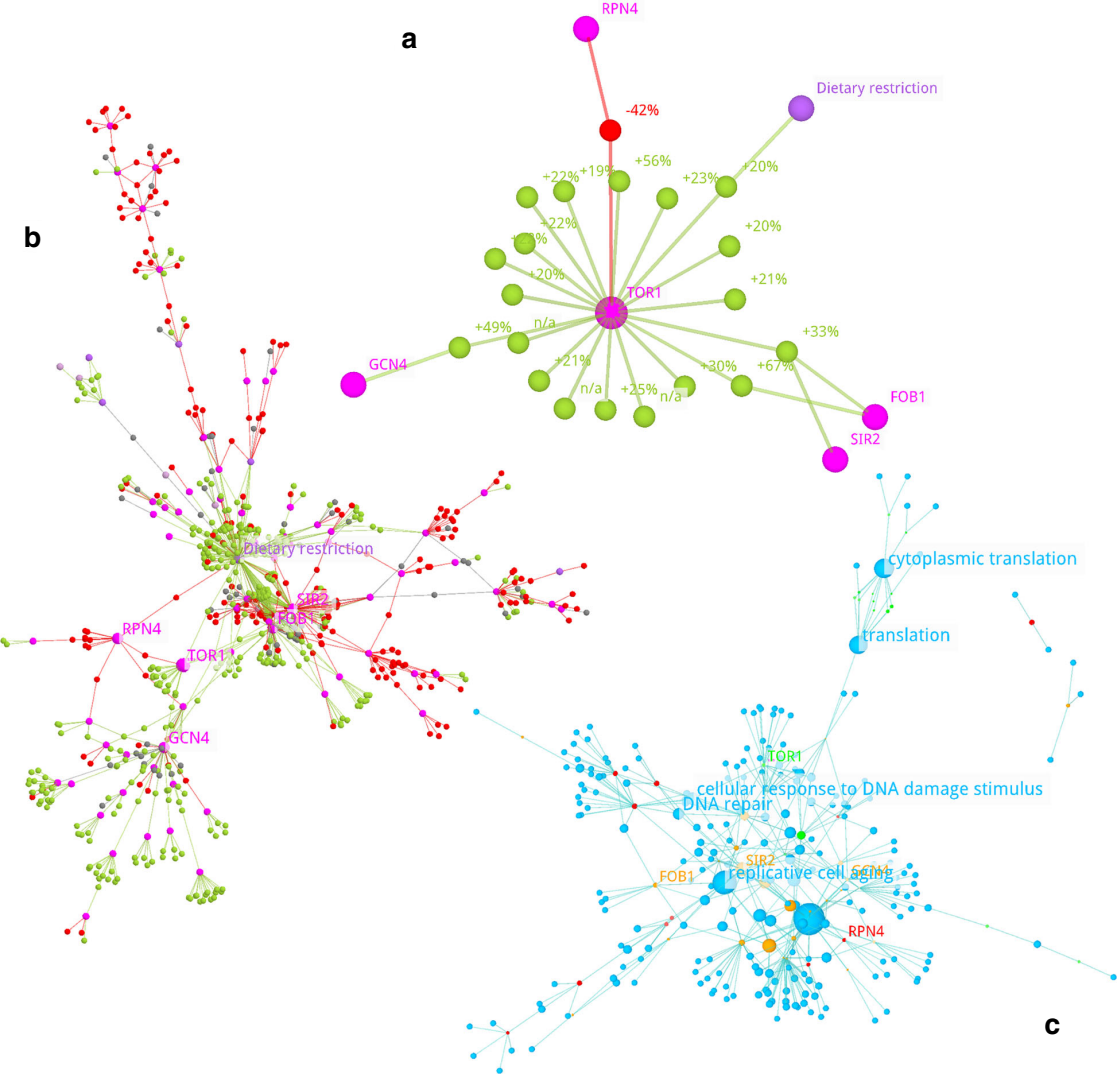
*TOR1* AF/LO Networks. **a** Basic *TOR1* AF/LO network containing only LOs involving the gene *TOR1* from *S. cerevisiae* and all other AFs involved directly in these LOs. LO nodes are labelled with the lifespan change value, if available. “n/a” indicates a missing value. The qualitative lifespan effect is also encoded in the LO node and edge colour, according to the colour scheme below. **Network size:** 26 nodes, 26 edges; **Layout calculation:** 3dFL algorithm with standard parameters; **b** Complete *TOR1* AF/LO network containing also all indirectly connected AFs and LOs in addition to those from the basic network in part **a**. The AFs from the basic network are labelled with their name. **Network size:** 718 nodes, 933 edges; **Layout calculation:** FMMM algorithm with standard parameters; **c***TOR1* network from part **b** augmented by GO process term nodes. AFs without assigned GO process terms were removed. The LOs were not included, but the information about the qualitative changes was retained in a summarised form, encoded in the AF node colours, according to the colour scheme shown below. The size of the GO process term nodes increases proportional to the number of linked genes (number of incoming edges). **Network size:** 284 nodes, 420 edges; **Layout calculation:** FMMM algorithm with standard parameters. The two subnetworks at the top left were moved manually after the export as PNG file; Color scheme (**a, b**):  AF - gene,  AF - compound,  AF - other factor,  LO - increased lifespan,  LO - decreased lifespan,  LO - unchanged lifespan Color scheme (**c**):  AF - linked to LOs with increased lifespan (opaque: only, transparent: ≥80%),  AF - linked to LOs with decreased lifespan (opaque: only, transparent: ≥80%),  AF - linked to LOs with highly mixed lifespan changes (increased and decreased >20%),  GO - process term

The direct neighbourhood example described here is focused on the AF *TOR1* from *S. cerevisiae* (Fig. 4a). The gene belongs to the TOR signalling pathway, which has been shown to regulate lifespan across multiple species (*S. cerevisiae, C. elegans, D. melanogaster*, and *M. musculus*), as part of the TORC1 complex [35]. The direct neighbourhood consists of twenty LOs for *TOR1* and five additional AFs (Dietary restriction, *FOB1*, *GCN4*, *RPN4*, *SIR2*) that are involved in these LOs.

The layout was calculated with the 3dFL algorithm. Different AF types are colour-coded: magenta indicates genes, dark purple indicates other factors, and light purple indicates chemical compounds (not present here). Each LO node is labelled with the lifespan change value, if available (n/a indicates a missing value).

Hovering over an LO node in the viewer provides additional information on the lifespan experiment. For this particular network all experiments were designed for the inactivation of *TOR1*. Those 15 LOs that are not connected to any other AF show a mean lifespan increase of 19% up to 56% for the inactivation of *TOR1*. In combination with the inactivation of the genes *FOB1*, *GCN4*, *SIR2* are combined with dietary restriction, the lifespan was increased in the range of 19% up to 67%. For an inactivation of the gene *RPN4* and *TOR1*, a lifespan decline of 42% was observed.

By focusing on those LOs that involve *TOR1* directly, the direct neighbourhood enables a quick overview of all 20 lifespan experiments involving it. The compact 3D view as a network can reveal the lifespan changes in combination with the other ageing factors more quickly than the large LO table for *TOR1* available in AgeFactDB. Due to the rather small size with 26 nodes and 26 edges a 2D view is already helpful too.

#### Example 2: complete neighbourhood

The direct neighbourhood network of the AF *TOR1* from the previous example was iteratively extended to the complete neighbourhood case. Figure 4b shows the complete neighbourhood graph of *TOR1*. It can be seen as the largest connected subgraph including *TOR1* and all directly or indirectly connected LOs and AFs for *S. cerevisiae*. The complete network consists of 718 nodes (78 AFs, 640 LOs) and 933 edges. The layout was built using the FMMM algorithm.

The extended graph reveals new information on *RPN4*. All experiments that included this gene led to a decreased lifespan. For *GCN4*, *FOB1*, and *SIR2*, the other direct neighbours of *TOR1*, there were observed decreased as well as increased lifespans.

The inclusion of all directly or indirectly connected LOs and AFs into the network enables to get an overview of all ageing factors examined directly or indirectly with *TOR1*. It would be much more laborious to compile the same dataset from the tabular AgeFactDB data and it would result in a very large table. In contrast to example 1, the much larger network profits much more from the 3D view compared to a 2D view. To illustrate this, a 2D view of this network, generated with the popular 2D network viewer Cytoscape [25], is shown in Additional file 1: Figure S2 and a JANet stereo representation in Additional file 1: Figure S3. The advantage of the 3D view will become even more obvious comparing interactive views in JANet and Cytoscape.

#### Example 3: augmentation via annotation nodes

In the augmentation example shown here the direct neighbourhood network is augmented by allele type (AT) and citation (CI) nodes. The AT provides information about the experimental manipulation of a gene, for example deletion or overexpression. To increase the benefit of adding AT nodes, we unified the ATs according to Table 3. There are for example 12 different ATs which are unified to *loss of function*. The original AT information was kept as annotation of the LO nodes.

**Table 3:** Allele Type (AT) Unification

AT	AT count	Unified AT
Conditional restoration of Fgf23 activity in Fgf23 knockout mice	1	Gain of function
Gain of function	27	Gain of function
Knockin	2	Gain of function
Conditional knockout	1	Loss of function
Deletion	1013	Loss of function
Deletion / null	1366	Loss of function
Deletion in connective tissue	1	Loss of function
Deletion of a region	1	Loss of function
Dominant negative	27	Loss of function
Gene disruption	1	Loss of function
Knock-down	1	Loss of function
Knockdown	2	Loss of function
Knockout	67	Loss of function
Loss of function	1631	Loss of function
Null mutant	4	Loss of function
Non-null dominant	3	Mutation
Non-null recessive	93	Mutation
Non-null semi-dominant	13	Mutation
Dominant negative mutation	4	Mutation
Mutation	135	Mutation
Mutation in adults	1	Mutation
Mutations	45	Mutation
Increased dosage	1	Overexpression
Overexpression	138	Overexpression
Overexpression in cardiac and skeletal muscles	1	Overexpression
Overexpression in skin	1	Overexpression
Overexpression in stem and progenitor cells	1	Overexpression
Overexpression of the short isoform of p53 (p44)	1	Overexpression
Over-expression	381	Overexpression
Pharmacological overexpression (Superoxide dismutase/catalase mimetics)	1	Overexpression
RNA interference	561	RNA interference
RNA interference and deletion	1	RNA interference
RNA interference in adults	2	RNA interference
RNA interference post development	47	RNA interference
RNA interference, Knockdown	1	RNA interference
RNAi knockdown	1819	RNA interference
Anti-sense RNA	1	Anti-sense RNA
Deletion / null + RNAi knockdown	1	Deletionnull + RNAi knockdown
Deletion / null + dominant negative	1	Deletion/null + Dominant negative
Deletion / null + normal	2	Deletion / null + normal
Deletion/null + over-expression	1	Deletion / null + over-expression
Epigenetic modification	2	Epigenetic modification
Gain of function + loss of function	1	Gain of function + loss of function
Germline ablation in daf-2 mutants	1	Germline ablation in daf-2 mutants
AT	AT count	Unified AT
Loss of function + RNAi knockdown	3	Loss of function + RNAi knockdown
Loss of function + normal	1	Loss of function + normal
Loss of function + over-expression	6	Loss of function + over-expression
Normal	31	Normal
Overexpression or mutations	1	Overexpression or mutations
Overexpression, mutations, deletion	1	Overexpression, mutations, deletion
Over-expression+ over-expression	2	Over-expression + over-expression
Over-expression+ unknown	2	Over-expression + unknown
RNA interference and mutations	1	RNA interference and mutations
RNA interference, Mutations	1	RNA interference, Mutations
Transient expression	1	Transient expression
Unknown	447	Unknown

The AT provides information about the experimental change applied to a gene, for example deletion or overexpression. To increase the benefit of adding AT nodes to networks the ATs were unified. There are for example 12 different ATs which are unified to *loss of function*. The upper part of the table, until the horizontal line, contains all unified ATs with at least 2 non-unified ATs. The AT count indicates the number of occurrences within AgeFactDB lifespan observations. The original AT information was kept as annotation of the LO nodes. The lower part of the table contains all otherATs, which were not unified

CI nodes provide information about the publication from which an LO was extracted, represented by the corresponding PubMed ID.

**Fig. 5:**
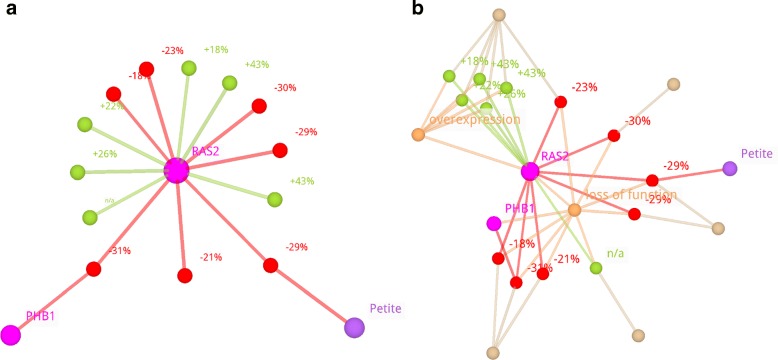
Basic *RAS2* AF/LO Network Augmented with AT and CI Nodes. Shown is the AF/LO network containing only LOs involving the gene *RAS2* from *S. cerevisiae* and all AFs involved in these LOs (basic *RAS2* network). LO nodes are labelled with the lifespan change value, if available. “n/a” indicates a missing value. The qualitative lifespan effect is also encoded in the LO node and edge colour, according to the colour scheme below. **a** There are 11 LOs were only the *RAS2* gene is involved. But the lifespan change values seem to be contradictory: 6 times an increased lifespan versus 5 times a decreased lifespan. **Network size:** 16 nodes, 15 edges; **Layout calculation:** 3dFL algorithm with standard parameters; **b** In addition to the nodes in part **a**, ATs and CIs from these LOs are included. ATs describe the experimental changes applied to genes, for example deletion or overexpression. They were unified according to Table 3. Citations resemble the publications from which the LOs were extracted, represented by the corresponding PubMed IDs. In contrast to the pure basic network in part A, most of the possible contradictions between the lifespan changes are resolved immediately. In all cases with reduced lifespan the *RAS2* gene was deactivated (AT: loss of function). In 5 of 6 cases with increased lifespan it was overexpressed instead (AT: overexpression). So the differences here fit to the expectation that overexpression of a gene has the opposite effect compared to loss of function. This leaves a single contradiction. One can see that the remaining contradictory LO was extracted from a different publication than the others. In general, this could be a hint that the experiments might have been performed under different conditions, which were not recorded during the extraction of the LO. **Network size:** 23 nodes, 44 edges; **Layout calculation:** 3dFL algorithm with standard parameters; Color scheme (**a, b**):  AF - gene,  AF - other factor,  LO - increased lifespan,  LO - decreased lifespan,  AT,  CI

Figure 5a displays the basic network of the gene *RAS2* from *S. cerevisiae*. It is homologous to members of the the mammalian RAS oncogene family, involved in the development of cancers [36]. There are 11 LOs where only the *RAS2* gene is involved. The corresponding lifespan changes seem to be contradictory: 6 times an increased lifespan versus 5 times a decreased lifespan. In Fig. 5b the basic network is augmented by AT and CI nodes. Here, most of the supposed contradictions are resolved immediately. In all cases with reduced lifespan the *RAS2* gene was deactivated (AT: loss of function). In 5 of 6 cases with increased lifespan it was overexpressed instead (AT: overexpression). So the differences here fit to the expectation that overexpression of a gene has the opposite effect compared to loss of function. It can be seen that the remaining contradictory LO was extracted from a different publication than the others. In general, this could be a hint that the experiments might have been performed under different conditions, which were not recorded during the extraction of the LO. But in this case, we could not resolve the contradiction by studying the two publications.

This example demonstrates how helpful it can be to include annotation information like ATs and CIs as additional nodes. Potential inconsistencies can be resolved quickly, without having to look up and remember the annotations individually for each node. Because the annotation nodes influence the network layout they can also help to identify quickly characteristics of the examined ageing factors. For *RAS2* such a characteristic is, that it seems to be a longevity promoting gene, meaning that its overexpression prolongs life while its inactivation shortens life.

#### Example 4: augmentation combined with data transfer

In this example the techniques *Augmentation via Annotation Nodes* and *Data Transfer* are combined within the following 2 steps.

Step1: The complete neighbourhood network of *TOR1* from Fig. 4b was augmented with Gene Ontology (GO) terms [28]. The resulting intermediate visualisation is shown in Additional file 1: Figure S4.

The augmentation increased the network size by about thirty percent, from 718 to 947 nodes (78 AFs, 640 LOs, 229 GO terms) and from 933 to 1353 edges. A stereo representation of the intermediate visualisation can be found in Additional file 1: Figure S5.

Step2: The *Data Transfer* from the LOs to the AFs in the second step compensates the increased network complexity by allowing to remove all 640 LO nodes (Fig. 4c). Here, the average observed lifespan changes are indicated by the colours of AF nodes. Genes, for which all connected LOs increased or decreased the lifespan, are coloured in green or red. Genes, for which the effects of at least eighty percent of the LOs are going into the same direction, are coloured translucent green or red. Genes with even more heterogeneous LOs are coloured in orange.

The blue annotation nodes symbolize GO terms of molecular processes. The size of the nodes increases proportional to the number of linked genes (number of incoming edges).

The annotation nodes group connected components close to each other. While GO terms connected with several genes tend to build clusters within the network, GO terms related to a single gene build satellites at the outside of the network.

Some of the GO terms are assigned to a larger number of AFs, indicated by the node size. The labelled GO terms *replicative cell aging* (connected to 14 AFs), *cellular response to DNA damage stimulus* (connected to 6 AFs), and *DNA repair* (connected to AFs) reveal therefore a connection of many AFs in the network to cell ageing and DNA repair processes playing an important and widely accepted role in ageing.

This example demonstrates on one hand the combination of two visualisation techniques. On the other hand it shows how the increase in network size and complexity by the inclusion of GO annotation nodes can be compensated. And the stereo representation in Additional file 1: Figure S3 provides a good impression of the benefit of a 3D network layout.

### JANet use cases

We provide use cases for the application of JANet. In the first use case, JANet is utilised for analysing a set of differentially expressed genes. In the second use case we demonstrate how JANet can be used for the identification of novel candidate genes related to ageing.

#### Use case 1: analysis of differentially expressed genes

JANet can be used to inspect user-specified gene lists within the LO networks extracted from AgeFactDB.

As an example we analyse differentially expressed genes taken from a study on the effect of D-Glucosamine (GlcN) on the lifespan of nematodes and ageing mice by Weimer et.al. [37]. The study comprises RNA-seq data of 12 *C. elegans* samples and 12 *M. musculus* samples. For each species, 6 samples were treated with GlcN supplementation; the other samples remained untreated. Data are available in the NCBI Gene Expression Omnibus (GEO) database [38] under accession GSE54853.

In their experiments, Weimer et al. identified 293 differentially expressed genes in mouse liver and 1272 genes in *C. elegans*. We analyse the combined list with 1565 genes.

Figure 2c illustrates how the analysis is started within JANet:

1. Copy / paste the list of differentially expressed genes into the tab “Import Gene List” (The viewer needs either the ID from the NCBI Gene database [39] or the gene symbol plus species name to be able to match a gene to an AF or putative AF.)
2. Start the analysis by clicking the “Start analysis” button.

The results are presented in a table containing the genes matching an AgeFactDB gene. Five entries of 157 matching gene entries are shown in Fig. 2d. For each gene the information provided by the user (NCBI Gene ID or gene symbol plus species name) and the corresponding information within AgeFactDB is shown. The table also provides the ageing relevance evidence type, characterising a matching gene as an ageing factor (“experimental evidence”) or a putative ageing factor (“homology analysis”). For putative ageing factors the homologous non-putative ageing factors are specified.

**Fig. 6:**
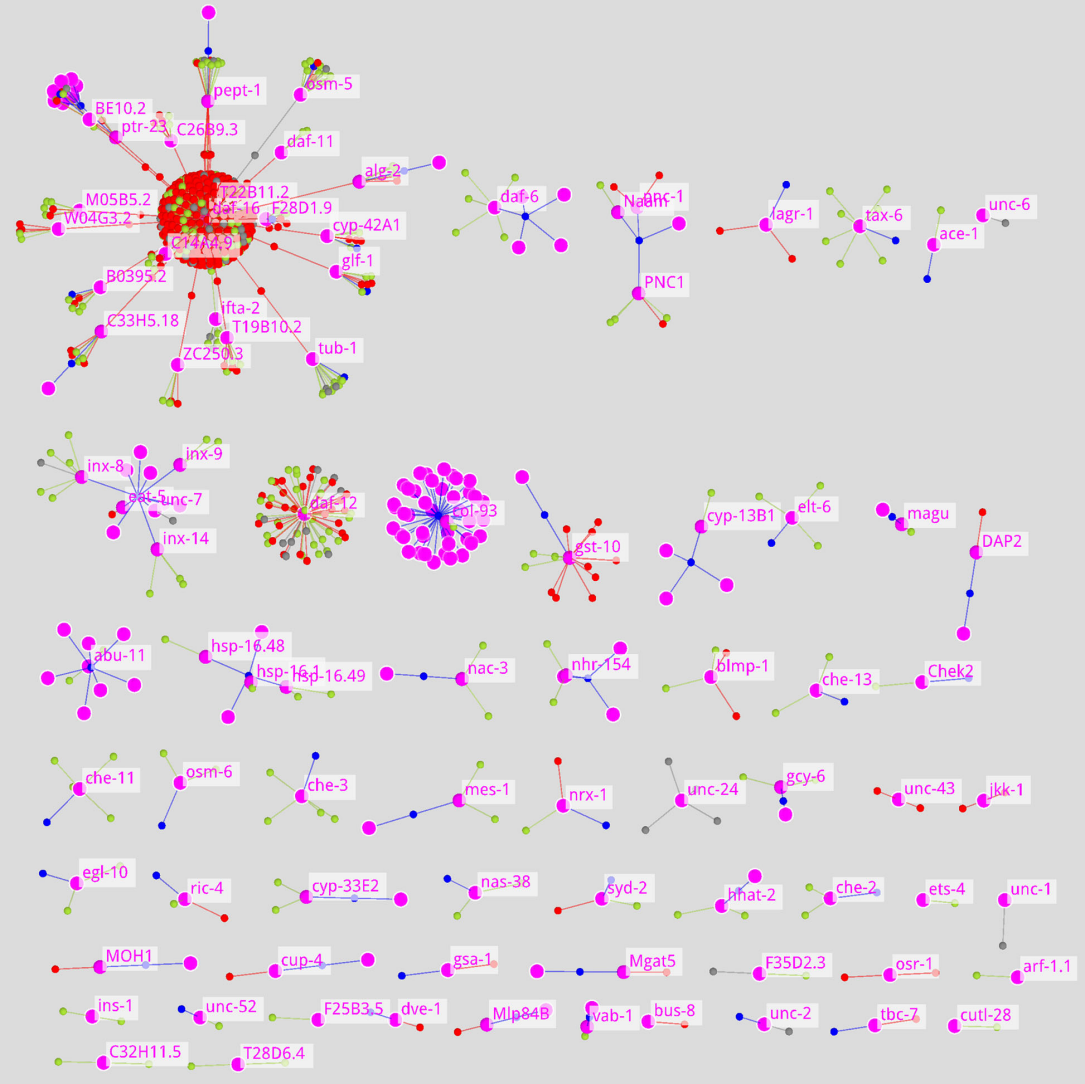
Differentially Expressed Genes Matched to AF/LO Subnetworks in a Tiled View. Shown are all 60 AF/LO subnetworks containing at least one gene from the list of differentially expressed genes [37]. The homology observations are included in the subnetworks without genes that are not from the list of differentially expressed genes. The 151 differentially expressed genes are marked by a halo. Genes with experimental ageing relevance evidence are labelled with their gene symbol. The qualitative lifespan effect is encoded in the LO node and edge colour, according to the colour scheme below. **Network size:** 952 nodes, 898 edges; **Layout calculation:** FMMM algorithm with standard parameters; **Color scheme:**  AF - gene,  AF - compound,  AF - other factor,  LO - increased lifespan,  LO - decreased lifespan,  LO - unchanged lifespan,  observation - homology analysis,  differentially expressed gene (gene of interest)

A visualisation of the results is presented in Fig. 6. It provides an overall impression on the differentially expressed genes and their fit into the LOs in AgeFactDB. For each matched differentially expressed gene the LO network is shown (FMMM). Only matching genes or their homologues with experimental evidence are included. Other genes involved in the LOs were excluded to provide a clearer overview.

The network also includes the observations from the homology analysis. Again, non-matching genes belonging to the homology groups were excluded. The genes from the user specified list are marked by a halo. 151 genes with LOs are given in the graph.

The first subnetwork at the top left is very large and looks rather different. It is centred around the ageing factor *daf-16* from *C. elegans*. For this gene many LOs are available (384). Most of them are concentrated in the sphere-like structure at the centre of the subnetwork. It was tested in combination with many other genes. A large number of AFs (17) and putative AFs (11) match to differentially expressed genes.

The second subnetwork is centred on the AF *col-93* from *C. elegans*. It only contains a single LO and a homology observation with a large homology group of 42 putative AFs, arranged in a sphere-like structure. The AF *col-93* is not in the user specified list. The third subnetwork centred on *daf-12* contains no other genes but only 60 LOs arranged in a sphere-like structure.

The other subnetworks have a rather similar structure: They consist of 1 to 5 AFs with a at most 13 LOs, 7 putative AFs, and 1 homology analysis observation. Several of the homology analysis observations are not connected to any putative AFs. This means that none of the other members of the homology group are on the user specified list.

Individual networks can be generated for each of the matching genes in the user specified list. An interactive table (Fig. 2d) can be used to narrow the number of AFs. Putative AFs are marked by a background colour in the interactive table.

The gene *Gstp2* (*Mus musculus*) from the user specified list is an example for a putative AF in AgeFactDB. For this type of AF, no LOs are available in the database. The list from Fig. 2d provides the homologous gene *gst-10* from *C. elegans* for which LOs exist. Networks for *gst-10* can be constructed via a dropdown list (Fig. 2a). The result is shown in Additional file 1: Figure S6.

In contrast to the overview network (Fig. 6), all AFs involved in the LOs are included. The network is augmented with AT and CI nodes. The numbers at the LO nodes indicate the lifespan change. It can be seen that overexpression of the *gst-10* resulted in an increased lifespan by about 20 percent, while reducing the expression by RNA interference resulted in a decreased lifespan by about 12 percent. It can also be seen that this data was gathered from the experiments reported in 3 publications.

This use case demonstrates how easy you can identify AFs within a large list of genes with JANet. The overview network provides a compact view of all LOs available for these ageing factors. The tiling of disconnected subnetworks separates AFs studied more extensively in combination with other AFs from those studied separately. Individual genes can also easily be looked at in more detail by building individual subsets.

#### Use case 2: candidate gene identification

JANet can be used for a de novo candidate gene identification on the basis of AgeFactDB. The following section provides a show case for a possible selection process for *C. elegans*. The task will be the identification of new promising candidates for ageing-related genes that are not yet included in AgeFactDB.

An overview of the candidate gene selection process is given in Fig. 7. It consists of nine major steps. For each step there is also an enlarged image available as additional file (Additional file 2: Figures S7–S15).

**Fig. 7:**
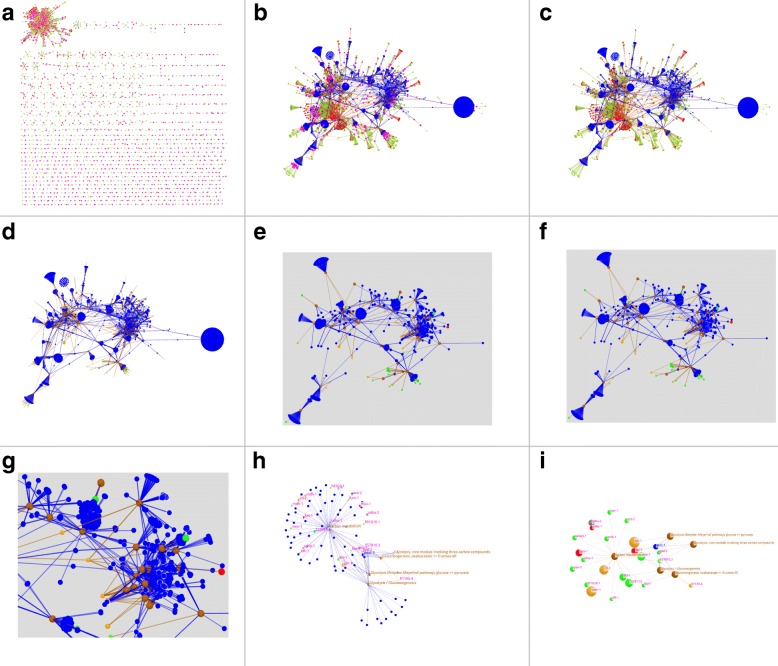
Candidate Gene Identification - Overview. Shown is an overview of the visualisation strategy to identify new candidate genes of *C. elegans* for inclusion in the database AgeFactDB. For each part of this figure there is also a larger version available, as an individual figure with a more detailed description (Additional file 2: Figures S7–S15). In the combined colour scheme below it is indicated which colours are applicable to which part. The layout was calculated in step 1 and 2 using the FMMM algorithm. Throughout step 3 to 8 the layout from step 2 was used, to facilitate tracking the changes. Instead of changing the layout, nodes were hidden, or node attributes like colour or size were changed during these steps. In step 9 a new layout was calculated using the FR algorithm. **a** Step 1: the complete AF/LO network of *C. elegans* with 675 subnetworks as starting point (Additional file 2: Figure S7); **b** Step 2: augmentation with KEGG pathway and gene nodes, based on cross-linking information from the BioSystems database (Additional file 2: Figure S8); **c** Step3: visual transfer of lifespan change information from LO nodes to AF nodes (Additional file 2: Figure S9); **d** Step 4: reduction of network complexity by hiding all LO nodes (Additional file 2: Figure S10); **e** Step 5: first reduction of candidate gene number by applying a minimum lifespan change limit (Additional file 2: Figure S11); **f** Step 6: second reduction of candidate gene number by selecting only genes connected to at least 6 KEGG pathway nodes; final selected candidate genes are marked by a halo (Additional file 2: Figure S12); **g** Step 7: zoom into the area with most of the candidate genes; Table 4 lists the final selected candidate genes and their literature analysis results (Additional file 2: Figure S13); **h** Step 8: focus on candidate gene *enol-1* by zooming and selective node display (Additional file 2: Figure S14); **i** Step 9: visualisation of the basic pathway network of candidate gene *enol-1*, augmented by AFs with data transferred from their LOs (Additional file 2: Figure S15); **Color scheme:**  AF - gene (**a**,**b**),  AF - compound (**a**),  AF - other factor (**a**),  AF - LOs with increased lifespan (opaque: only, transparent: ≥80%; **c**,**d**,**e**,**f**,**g**,**h**,**i**),  AF - LOs with decreased lifespan (opaque: only, transparent: ≥80%; **c**,**d**,**i**),  AF - LOs with highly mixed lifespan changes (increased and decreased >20%; **c**,**d**,**e**,**f**,**g**,**h**,**i**),  LO - increased lifespan (**a**,**b**,**c** small),  LO - decreased lifespan (**a**,**b**,**c** small),  LO - unchanged lifespan (**a**,**b**,**c**),  KEGG pathway (**b**,**c**,**d**,**e**,**f**,**g**,**h**,**i**),  gene (from pathway and not in AgeFactDB; **b**,**c**,**d**,**e**,**f**,**g**,**h**),  selected candidate gene (**f**,**g**,**h**,**i**)

Step1: We start our screening with the inspection of the complete LO network of *C. elegans* (step 1, Fig. 7a and Additional file 2: Figure S7). It consists of 965 ageing-related genes and 4265 LOs and is divided into 676 disjunct subnetworks.

Step 2: The LO network is augmented by the introduction of pathway nodes that represent KEGG pathways [29] specific for *C. elegans*. These pathways were extracted from the BioSystems database [40], based on cross-linking information to AFs. The other gene components of a KEGG pathway are introduced as additional gene nodes, linked also to the pathway node. AFs unconnected to a pathway node are removed. The resulting network is shown in Fig. 7b and Additional file 2: Figure S8. This step reduced the numbers of ageing-related genes (324), LOs (2159) and disconnected subnetworks (1). A number of 2619 genes and 161 KEGG pathways were added. Linked to known AFs via common KEGG pathways, the additional genes can be seen as an initial set of candidates for ageing-related genes.

Step 3: A summary of the LO information was transferred to the AF nodes in preparation for reducing the complexity of the network (Fig. 7c and Additional file 2: Figure S9). This means that the LO node colours, indicating lifespan increase or decrease, from all LOs connected to an AF were transformed into a new colour for the AF node. The transformation was done by the following scheme:

- only red LOs → opaque red AF
- ≥80% red LOs → translucent red AF
- only green LOs → opaque green AF
- ≥80% green LOs → translucent green AF
- >20% red and >20% green LOs → orange AF

Step 4: The complexity of the network was reduced by hiding all 2159 LO nodes (Fig. 7d and Additional file 2: Figure S10).

Step 5: In order to focus on the most relevant AFs, the visible network was restricted to those AFs with a maximal lifespan change of at least 100%.

**Table 4:** *C. elegans* candidate genes

Gene symbol	NCBI gene ID	Ageing relevance evidence
*acdh-7, acdh-8*	181758 173979	Expression of human homologous gene *ACADM* decreases over age in skin [45].
*aco-1*	181324	Mutant animals lacking genes *aco-1* and *ftn-1* show significant reduced lifespan upon iron stress, while N2 and *ftn-2* animals show no difference. The results suggest that *ftn-1* and *aco-1* are transcriptionally regulated by iron and are important for iron homeostasis affecting lifespan upon iron stress conditions in *C. elegans* [42].
*alh-4*	179026	Gene *alh-4* is involved in determination of adult lifespan [46].
*acs-3*	178677	*acs-3* mutants exhibited a significantly shorter lifespan on *E. coli* OP50 (12.8 days), as compared to WT animals (17.9 days) [52].
*acs-4*	176005	RNAi of *acs-4* resulted in a 6.1% increase in lifespan compared to wildtype [53].
*acs-17*	180859	Many *“dod”* genes are responsible for the longevity of *daf-2* mutants. The *“dod”* genes that could previously be identified as life span regulating genes include among others *dod-9/acs-17* [54, 55].
*alh-5 alh-12 sodh-1 sodh-2*	178680 176056 179627 179628	Metabolic fingerprint studies with long-lived mutants *daf-2* and *eat-2* showed strong upregulation of enzymes involved in alcohol fermentation including *alh-5, alh-12, sodh-1* and *sodh-2* [56].
*dbl-1*	179068	*C. elegans* mutants with a loss-of-function in *dbl-1* showed reduced lifespan [57].
*ech-6*	176376	Metabolism of short-chain organic acids (e.g. gene *ech-6*) is upregulated in states of impaired IGF-1 signalling in a *daf-2* mutant strain with extended lifespan [47].
*ech-9*	184065	The top five overrepresented categories yielded by the comparative expression analysis in three *ets-4* deletion strains with increased lifespan include fatty acid metabolic process genes (*acdh-2*, *ech-9*, and *C48B4.1*) [48].
*enol-1*	174423	Compared to wild type strain N2 the RNAi knockdown of gene *enol-1* reduced the mean lifespan by 11-14%. Compared to *eat-2* knockout strains it reduced the mean lifespan by 15-19% in strain eat-2(ad1116) and by 28% in strain eat-2(ad465) [41].
*F08F8.7*	176043	*F08F8.7* was found to be upregulated in long-living mutant *daf-2* in comparison to N2 [56].
*F54C8.1*	186222	Concerning branched-chain amino acid metabolism, *F54C8.1* was upregulated in the long-lived mutant *daf-2* [56]
*F59F4.1*	181668	Gene *F59F4.1* was linked to Parkinson’s disease by screens involving alpha-synuclein. In alpha-synuclein expressing nematode lines age dependent degeneration of dopaminergic neurons was observed [49].
*hacd-1*	178638	Gene *hacd-1* was linked to lifespan effects of germline mutants by transcriptome analysis [50].
*idhg-2*	172430	Energy metabolism seems to be differentially regulated in long-lived mutants compared to N2. Particularly *idhg-2* was upregulated in *daf-2* mutants [56].
*mek-1*	181004	The lifespan extending effect of intermittent feeding (IF) of 74.9% in wiltype strain N2 is reduced to 21.7% in the *mek-1* loss-of-function strain ks54 [58].
*pfk-1.2*	179335	Long-living mutants show upregulation of *pfk-1.2* a gene encoding for one of the key enzymes of glycolysis [56].
*pkc-2*	181166	Knockout of gene *pkc-2* decreased the mean lifespan significantly by 3–5% at 15°C and compensated for the short-lived phenotype of *trpa-1* mutant worms at 20°C. Overexpression of gene *pkc-2* increased the mean lifespan significantly by 3–6% at 20°C [43].
*plc-3*	174586	The youthful swimming phenotype of *let-23 (gf)* mutant was suppressed by RNAi knockdown of *plc-3* and *itr-1* [59].
*pyk-1*	172744	Compared to wildtype strain N2 the RNAi knockdown of gene *pyk-1* reduced the mean lifespan by 19–21%. And compared to *eat-2* knockout strain eat-2(ad1116) it reduced the mean lifespan by 19–29% [41].
*R04A9.7*	3565956	The traditional Chinese medicine Gengnianchun (GNC) prolongs the lifespan of *C. elegans*. Comparison of genome-wide transcriptional profiling of untreated and GNC treated worms at day 22 showed that GNC downregulated the expression of R04A9.7 [60].
Gene symbol	NCBI gene ID	Ageing relevance evidence
*rbx-1*	179358	RNAi of *rbx-1* results terminal stage arrest during embryogenesis[61].
*sdhd-1*	174692	Extension of lifespan in *gas-1 (fc21)* was observed after *sdhc-1* and *sdhd-1* knockdown [62].
*skr-9 skr-14 sma-4*	178494 178839 175815	Wnt signaling is highly involved in the aging process of *C. elegans* with a shifting dynamic. From L4 to D6 Wnt signalling is upregulated and from D6 to D15 it is down-regulated. Eight genes including *skr-9, skr-14*, and *sma-4*, which are involved in the Wnt signalling, were significantly up-regulated from L4 to D6 and down-regulated from D6 to D15 [63].
*sucg-1, sucl-2*	177555 175252	The decrease of female reproductive senescence is a hallmark of ageing. The inactivation of *sucg-1* or *sucl-2* by RNAi extends the reproductive lifespan [64].
*T02G5.7*	3565206	The gene *kat-1*, a parolog of gene *T02G5.7*, is included as AF AF_006931 in AgeFactDB [51].
*tpi-1*	174844	RNAi knockdown of gene *tpi-1* reduced the the mean lifespan by 8% and the maximum lifespan by 10% [44].

Candidate genes obtained by the candidate gene selection strategy summarised in Fig. 7. Results of the literature search for ageing relevance evidence. No specific ageing relevance evidence was found for the following genes: *acox-1*, *acox-3*, *acox-5*, *acs-2*, *acs-13*, *acs-15*, *acs-16*, *acs-18*, *acs-19*, *acs-23*, *agxt-1*, *aldo-2*, *alh-8*, *alh-9*, *B0272.3*, *C30H6.7*, *C44H4.6*, *C50D2.7*, *D2063.1*, *dlst-1*, *ech-8*, *F08A8.4*, *F11F1.1*, *F44E5.4*, *F44E5.5*, *got-1.3*, *got-2.1*, *got-2.2*, *gstk-1*, *hsp-70*, *hxk-1*, *idhg-1*, *ist-1*, *pdhb-1*, *pfk-1.1*, *pgk-1*, *R03D7.5*, *R05F9.6*, *rpia-1*, *skr-4*, *skr-6*, *skr-10*, *skr-16*, *skr-17*, *skr-18*, *skr-21*, *suca-1*, *sucl-1*, *tpi-1*, *ugt-23*, *ugt-46*, *ugt-48*, *ugt-50*, *ugt-55*, *ugt-56*, *ugt-58*, *ugt-61*, *ugt-62*, *Y105E8B.9*, *Y43F4B.5*, *Y71G12B.10*

Only the corresponding connected pathways and additional genes are shown (Fig. 7e and Additional file 2: Figure S11). It consists now of 768 additional genes connected to 46 pathways.

Step 6: Based on the assumption that genes which are connected to AFs via multiple pathways are more likely to be ageing-related, promising candidates were highlighted. Only those genes connected to at least 6 pathway nodes were selected and marked by a halo (Fig. 7f and Additional file 2: Figure S12). This led to a final list of 95 candidate genes connected to 20 visible pathway nodes.

Step 7: A more detailed view was created by zooming in at the position with the largest number of highlighted candidate genes (Fig. 7g and Additional file 2: Figure S13).

Step 8: It is also possible to reinspect the network while focusing on a single candidate gene. As an example gene *enol-1* was used (Fig. 7h and Additional file 2: Figure S14). We revisited the network before the reduction of the pathway and gene nodes (step 4), while focusing on *enol-1*. All nodes were hidden that are not connected to the 7 pathway nodes which are connected to *enol-1*. The candidate gene node, the AF nodes, and the pathway nodes are labelled with their names.

Step 9: An optimized view was created for candidate gene *enol-1* by building a new subset and calculating a new layout (Fig. 7i and Additional file 2: Figure S15). The new subset contains *enol-1*, all KEGG pathway nodes connected to it, and all AFs connected to these pathway nodes. Like before, information from the LOs was transferred to the AFs and visualized by node colour and size. As you can see, *enol-1* is linked to 3 different types of pathways: biosynthesis, degradation and energy metabolism. The LOs were obtained in the context of energy metabolism [41].

For all 95 selected candidate genes obtained in step 6 we did a literature search. The results are summarized in Table 4. LOs not yet contained in AgeFactDB with a significant lifespan change are available for the 5 candidate genes *aco-1*, *enol-1*, *pkc-2*, *pyk-1*, and *tpi-1* [41–44]. Other ageing relevance evidence was found for the 8 other candidate genes *acdh-7*, *acdh-9*, *alh-4*, *ech-6*, *ech-9*, *F59F4.1*, *hacd-1*, and *T02G5.7* [45–51]. No specific ageing relevance evidence was found for the remaining 35 candidate genes.

In this use case JANet helped to find new candidate genes as AFs for AgeFactDB. By applying several of the proposed visualisation techniques we could select 95 promising candidate genes from the initial 2619 genes. The literature search revealed for 27% of the candidate genes that there is ageing-relevance evidence. This true positive rate suggests that it would be justified to include also others of the remaining 35 candidate genes into experimental analysis. Moreover, such an in-silico approach may also improve the curation of AF/LO databases.

## Conclusion

JANet provides a wide range of network visualisations for the analysis of lifespan observations. Integrating heterogeneous data from various sources, these networks allow a comprehensive overview of data from lifespan experiments and their dependencies. The investigations are linked via common components or external domain knowledge into network representations. This can generate interpretable patterns, which are recognisable by a human expert. These network representations can be seen therefore as a valuable addition to classic tabularized representations.

Interactive network manipulation allows replacing complex static queries with a visually guided search process. A life scientist can easily explore a network by merely changing the graph’s perspective. For example, zooming into a region of interest can reveal detailed information, which might be hidden at a broader scale. In this context, interactive 3D layouts allow a more extensive range of manipulations than classical 2D graphs. Providing more compact graph representations and 3D rotation, 3D arrangements allow reaching distant points much faster. The network itself can be reconfigured during the exploration. For example, LOs can be hidden or highlighted.

In our first use case, we show that a researcher can utilise the network visualisations of AgeFactDB to explore his/her experimental data. This type of analysis brings a set of candidate genes into the context of thousands of LOs. In this way, the single experiment gets highly connected to the ageing research field, which would be more laborious by a traditional literature or database screen. This general ability of network representations of reflecting existing knowledge and facilitating the analysis of experimental data can be useful in many other research areas.

## Additional files

Additional file 1 **Figure S1**: Large AF/LO Network Navigation in a 3D and 2D Network Viewer. **Figure S2**: The Complete TOR1 AF/LO Network as 2D View. **Figure S3**: The Complete TOR1 AF/LO Network as JANet Stereogram. **Figure S4**: The Complete TOR1 AF/LO Network Augmented with GO Process Term Nodes. **Figure S5**: A Stereogram of the Complete TOR1 AF/LO Network Augmented with GO Process Term Nodes. **Figure S6**: The Differentially Expressed Gene *Gstp2* Matched to the AF/LO Subnetwork of Gene *gst-10*. (PDF 15,960 kb)

Additional file 2 **Figure S7**: Candidate Gene Selection - Step 1. **Figure S8**: Candidate Gene Selection - Step 2. **Figure S9**: Candidate Gene Selection - Step 3. **Figure S10**: Candidate Gene Selection - Step 4. **Figure S11**: Candidate Gene Selection - Step 5. **Figure S12**: Candidate Gene Selection - Step 6. **Figure S13**: Candidate Gene Selection - Step 7. **Figure S14**: Candidate Gene Selection - Step 8. **Figure S15**: Candidate Gene Selection - Step 9. (PDF 8189 kb)

## Abbreviations

3dFL: 3D forced layout
AF: Ageing factor
AgeFactDB: JenAge Ageing Factor Database
AT: Allele type
CI: Citation
*C. elegans*: *Caenorhabditis elegans*
FR: Fruchterman-Reingold algorithm
FMMM: Fast multipole multilevel method
GEO: Gene Expression Omnibus
GO: Gene ontology
JANet: Jmol AgeFactDB network-viewer
LO: Lifespan observation
*M. musculus*: *Mus musculus*
*S. cerevisiae*: *Saccharomyces cerevisiae*
