# Supplementary material for: 3D Network exploration and visualisation for lifespan data
Source: BMC Bioinformatics. 2018 Oct 23;19:390. doi: 10.1186/s12859-018-2393-x (PMC6199797; doi:10.1186/s12859-018-2393-x)
Supplement: Supplementary file 1 — Figure S1: Large AF/LO Network Navigation in a 3D and 2D Network Viewer. Figure S2: The Complete TOR1 AF/LO Network as 2D View. Figure S3: The Complete TOR1 AF/LO Network as JANet Stereogram. Figure S4: The Complete TOR1 AF/LO Network Augmented with GO Process Term Nodes. Figure S5: A Stereogram of the Complete TOR1 AF/LO Network Augmented with GO Process Term Nodes. Figure S6: The Differentially Expressed Gene Gstp2 Matched to the AF/LO Subnetwork of Gene gst-10. (PDF 15,960 kb) [file 12859_2018_2393_MOESM1_ESM.pdf]

Additional Files

Additional File 1 — Large AF/LO Network Navigation in a 3D and 2D Network Viewer (navigation_3d_versus_2d.png)

Additional File 2 — The Complete TOR1 AF/LO Network as 2D View (tor1_complete-cytoscape_2d.png)

Additional File 3 — The Complete TOR1 AF/LO Network as JANet Stereogram (tor1_complete-janet_stereo.png)

Additional File 4 — The Complete TOR1 AF/LO Network Augmented with GO Process Term Nodes (tor1_with_go.png)

Additional File 5 — A Stereogram of the Complete TOR1 AF/LO Network Augmented with GO Process Term Nodes (tor1_with_go-stereo.png)

Additional File 6 — The Differentially Expressed Gene *Gstp2* Matched to the AF/LO Subnetwork of Gene *gst-10* (goi_example-gst_10-.png)

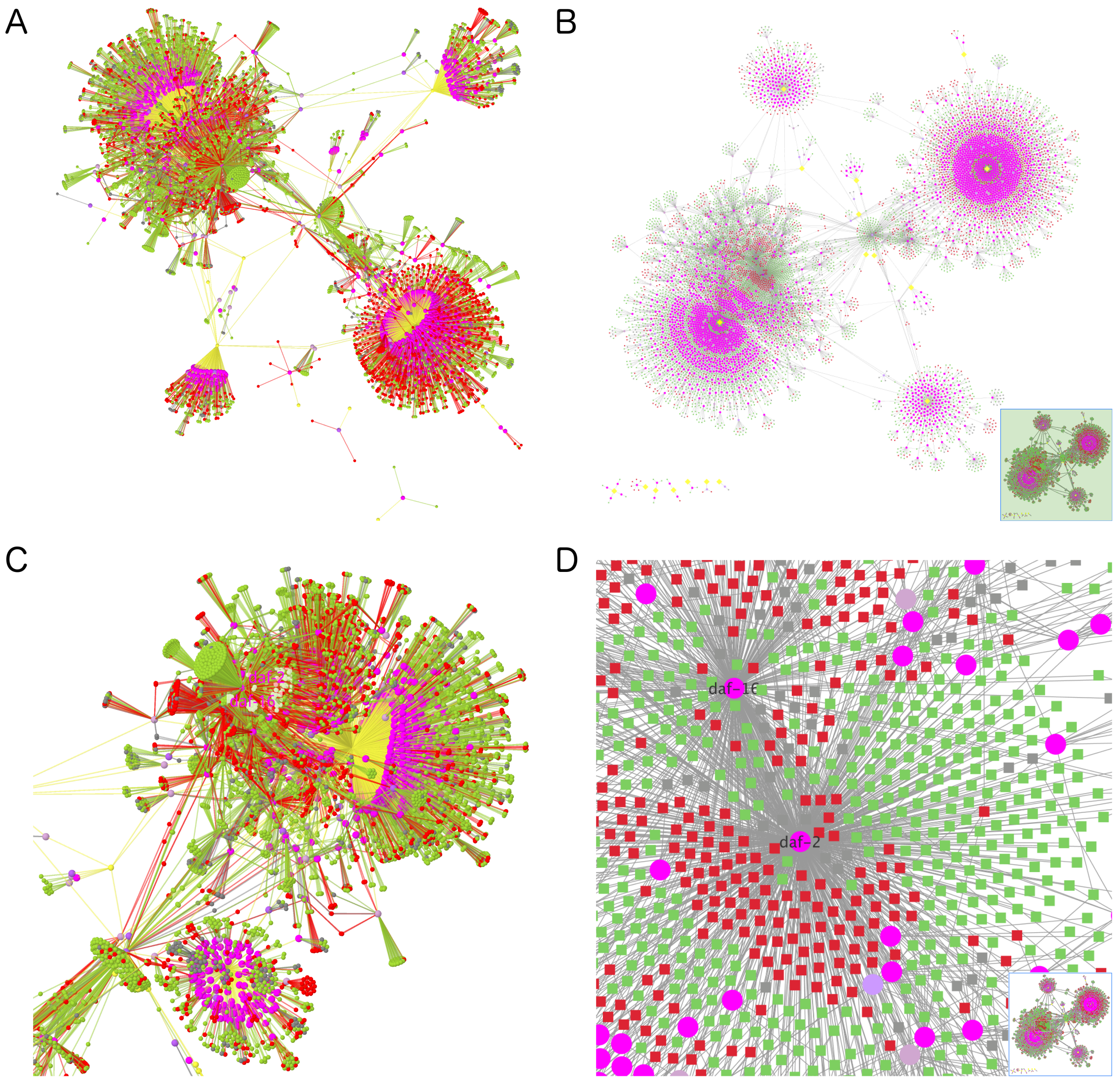

Additional File 1 Large AF/LO Network Navigation in a 3D and 2D Network Viewer. Navigation costs differ between 3D and 2D representations of larger networks. To illustrate this, a large AF/LO network containing all ageing factors (AFs) having lifespan observations (LOs), all LOs, and the corresponding species (SPs) is shown here as example.
Network size: 9560 nodes, 11534 edges; **3D layout calculation:** FMMM algorithm with standard parameters; **2D layout calculation:** Allegro Spring Electric from the Allegro Layout app in Cytoscape
Color scheme: AF - gene, AF - compound, AF - other factor, LO - increased lifespan, LO - decreased lifespan, LO - unchanged lifespan Species
A) Shown is a 3D view in JANet, containing the complete network. It can be explored quickly by rotation, zoom, and translation.
B) Shown is a 2D view in Cytoscape, including a navigation panel at the bottom right. It requires more space, resulting in a less detailed overview. It can be explored by zoom and translation.
C) This 3D view is focused on the 2 AFs *daf-2* and *daf-16* from *C. elegans*. A small zoom factor was already sufficient, reducing the navigation costs for changing the focus.
D) This 2D view is also focused on the 2 AFs *daf-2* and *daf-16* from *C. elegans*. A much larger zoom factor was needed here to achieve a similar level of detail as in C). This makes it more difficult to keep oriented about the current position inside the network, even with the help of the navigation panel, and increases also the navigation time.

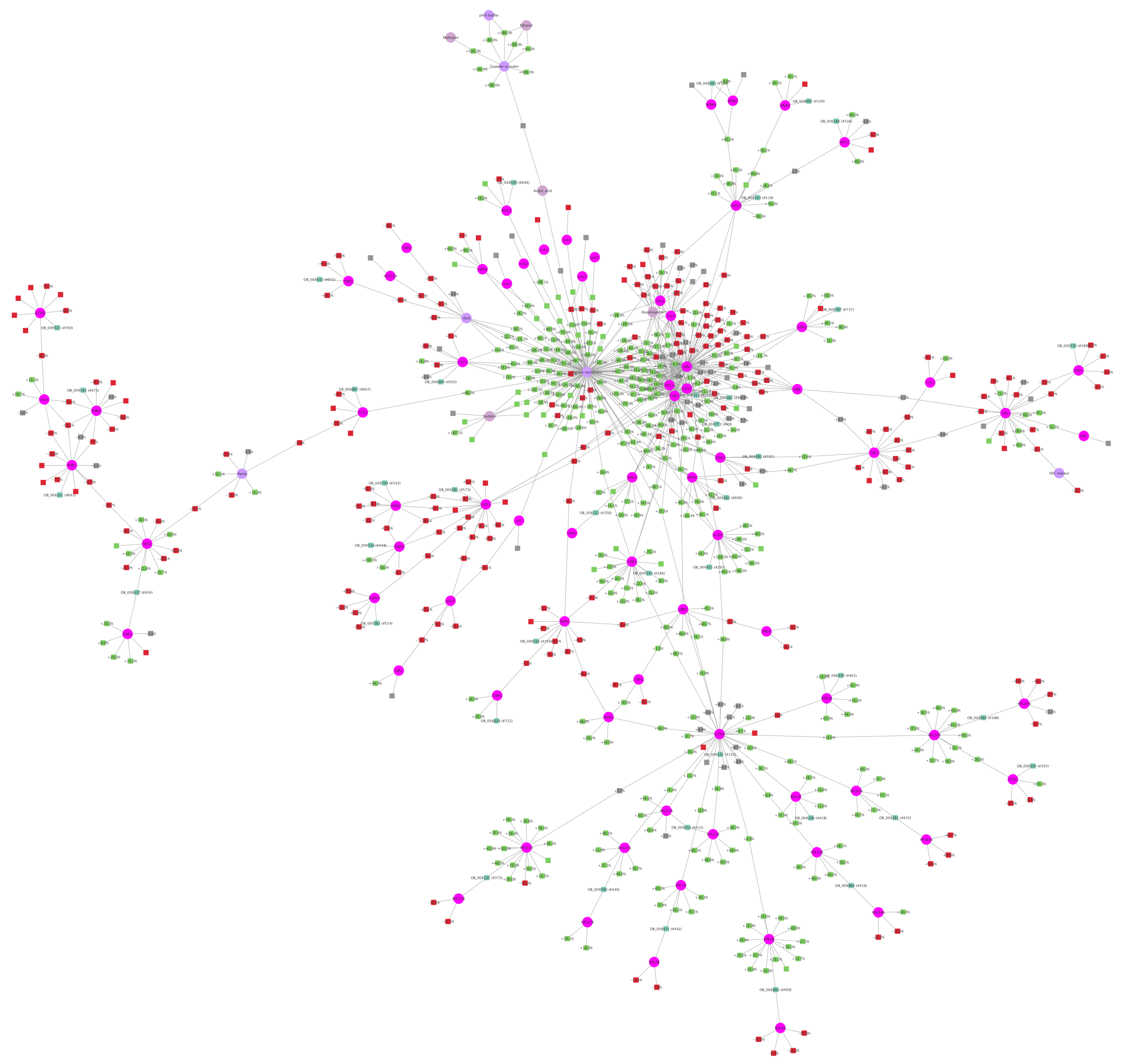

Additional File 2 The Complete TOR1 AF/LO Network as 2D View Shown is the AF/LO network containing the gene *TOR1* from *S. cerevisiae*, and all LOs and other AFs connected to it, also indirectly (complete network, Figure 4) as 2D view.
Network size: 718 nodes, 933 edges; **Layout calculation:** Allegro Spring Electric from the Allegro Layout app in Cytoscape
Color scheme: AF - gene, AF - compound, AF - other factor, LO - increased lifespan, LO - decreased lifespan, LO - unchanged lifespan

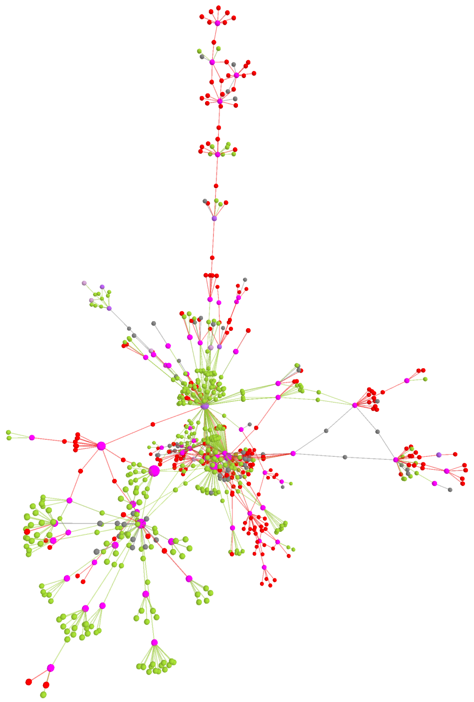

Additional File 3 The Complete TOR1 AF/LO Network as JANet Stereogram. Shown is the AF/LO network containing the gene *TOR1* from *S. cerevisiae*, and all LOs and other AFs connected to it, also indirectly (complete network, Figure 4) as JANet stereogram.
Network size: 718 nodes, 933 edges; **Layout calculation:** JANet 3D stereo view
Color scheme: AF - gene, AF - compound, AF - other factor, LO - increased lifespan, LO - decreased lifespan, LO - unchanged lifespan

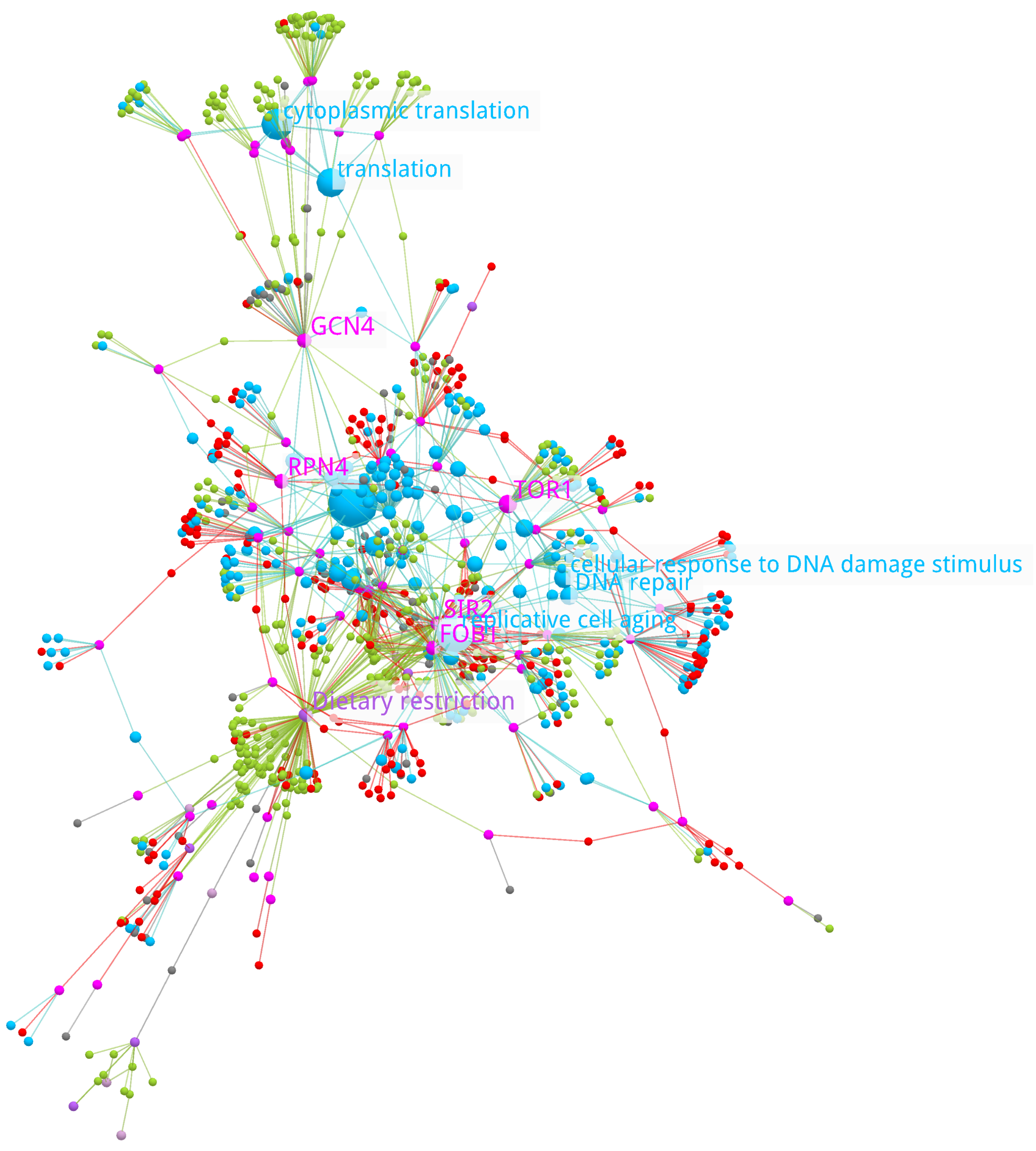

Additional File 4 The Complete TOR1 AF/LO Network Augmented with GO Process Term Nodes. Shown is the AF/LO network containing the gene *TOR1* from *S. cerevisiae*, and all LOs and other AFs connected to it, also indirectly (complete network). In addition, GO process terms assigned to the AFs are included. The qualitative lifespan effect is encoded in the LO node and edge colour, according to the colour scheme below. The size of GO term nodes reflects the number of their edges (degree). The AFs from the basic network (see Figure 4A) and selected GO terms with a high degree are labelled with their name.
Network size: 947 nodes, 1,383 edges; **Layout calculation:** FMMM algorithm with standard parameters;
Color scheme: AF - gene, AF - compound, AF - other factor, LO - increased lifespan, LO - decreased lifespan, LO - unchanged lifespan, GO process term

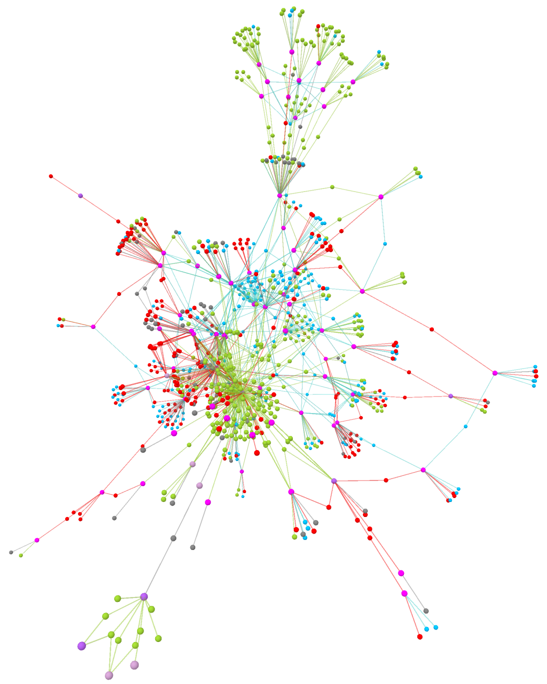

Additional File 5 A Stereogram of the Complete TOR1 AF/LO Network Augmented with GO Process Term Nodes Shown is the AF/LO network containing the gene *TOR1* from *S. cerevisiae*, and all LOs and other AFs connected to it, also indirectly (complete network). In addition, GO process terms assigned to the AFs are included. The qualitative lifespan effect is encoded in the LO node and edge colour, according to the colour scheme below. The size of GO term nodes reflects the number of their edges (degree). This figure is a stereogram version of the network in additional file 4.
Network size: 947 nodes, 1353 edges; **Layout calculation:** FMMM algorithm with standard parameters;
Color scheme: ● AF - gene, ● AF - compound, ● AF - other factor, ● LO - increased lifespan, ● LO - decreased lifespan, ● LO - unchanged lifespan, ● GO process term

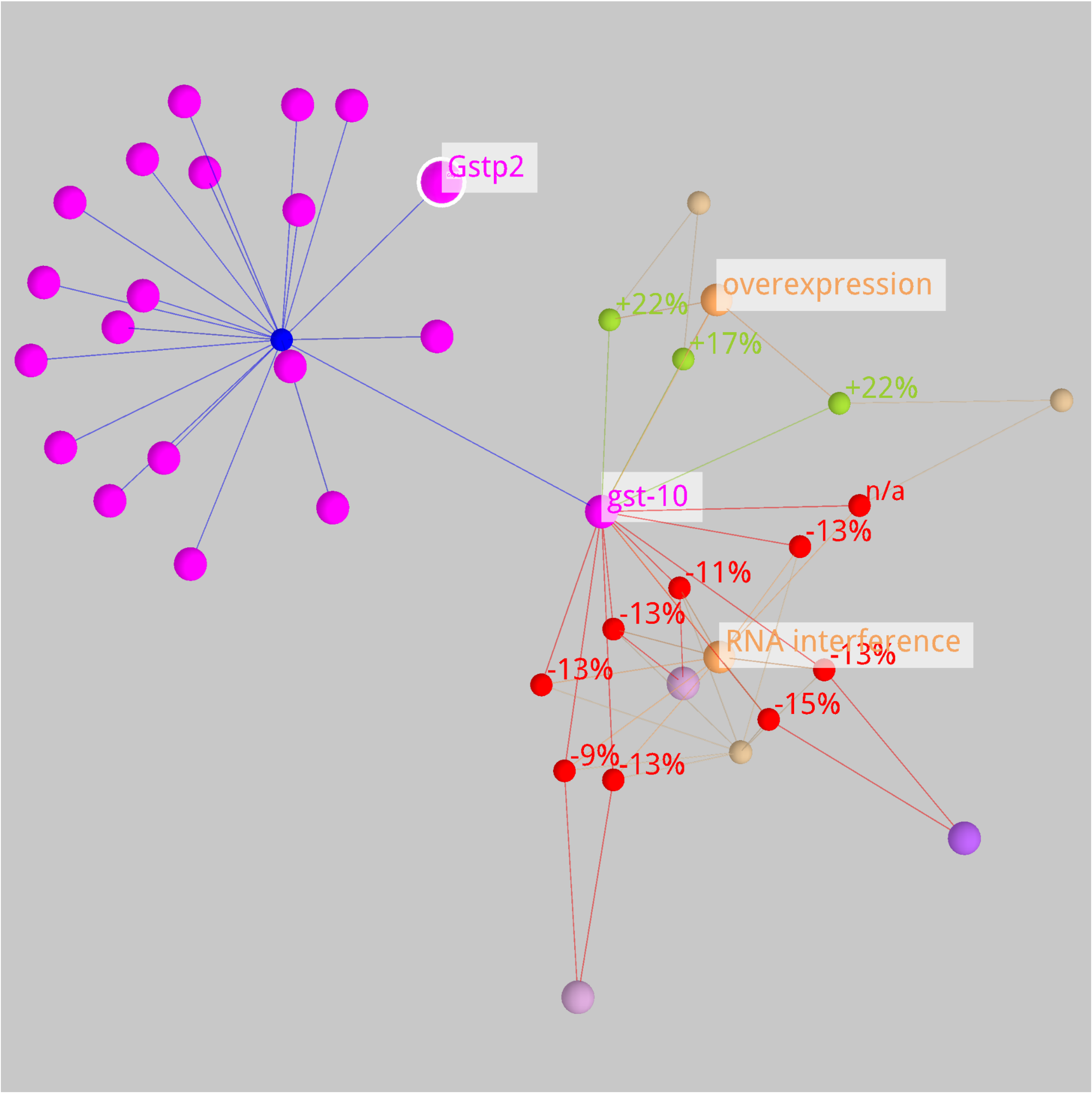

Additional File 6 The Differentially Expressed Gene Gstp2 Matched to the AF/LO Subnetwork of Gene gst-10 Shown is the AF/LO subnetwork of the AF gst-10 from *Caenorhabditis elegans* containing at least one gene of interest from the example lists of differentially expressed genes [37]. The homology observations are included in the subnetworks. The genes of interest are marked by a halo. The qualitative lifespan effect is encoded in the LO node and edge colour, according to the colour scheme below.
Network size: 41 nodes, 64 edges; **Layout calculation:** FMMM algorithm with standard parameters, afterwards the edge length of nodes with degree one was reduced to 40 percent;
Color scheme: AF - gene, AF - compound, AF - other factor, LO - increased lifespan, LO - decreased lifespan, LO - homology analysis, citation, AT - allele type, gene of interest
