# Supplementary material for: 3D Network exploration and visualisation for lifespan data
Source: BMC Bioinformatics. 2018 Oct 23;19:390. doi: 10.1186/s12859-018-2393-x (PMC6199797; doi:10.1186/s12859-018-2393-x)
Supplement: Supplementary file 2 — Figure S7: Candidate Gene Selection - Step 1. Figure S8: Candidate Gene Selection - Step 2. Figure S9: Candidate Gene Selection - Step 3. Figure S10: Candidate Gene Selection - Step 4. Figure S11: Candidate Gene Selection - Step 5. Figure S12: Candidate Gene Selection - Step 6. Figure S13: Candidate Gene Selection - Step 7. Figure S14: Candidate Gene Selection - Step 8. Figure S15: Candidate Gene Selection - Step 9. (PDF 8189 kb) [file 12859_2018_2393_MOESM2_ESM.pdf]

Additional File 7 — Candidate Gene Selection - Step 1
(select_candidates_1.png)

Additional File 8 — Candidate Gene Selection - Step 2
(select_candidates_2.png)

Additional File 9 — Candidate Gene Selection - Step 3
(select_candidates_3.png)

Additional File 10 — Candidate Gene Selection - Step 4
(select_candidates_4.png)

Additional File 11 — Candidate Gene Selection - Step 5
(select_candidates_5.png)

Additional File 12 — Candidate Gene Selection - Step 6
(select_candidates_6.png)

Additional File 13 — Candidate Gene Selection - Step 7
(select_candidates_7.png)

Additional File 14 — Candidate Gene Selection - Step 8
(select_candidates_8.png)

Additional File 15 — Candidate Gene Selection - Step 9
(select_candidates_9.png)

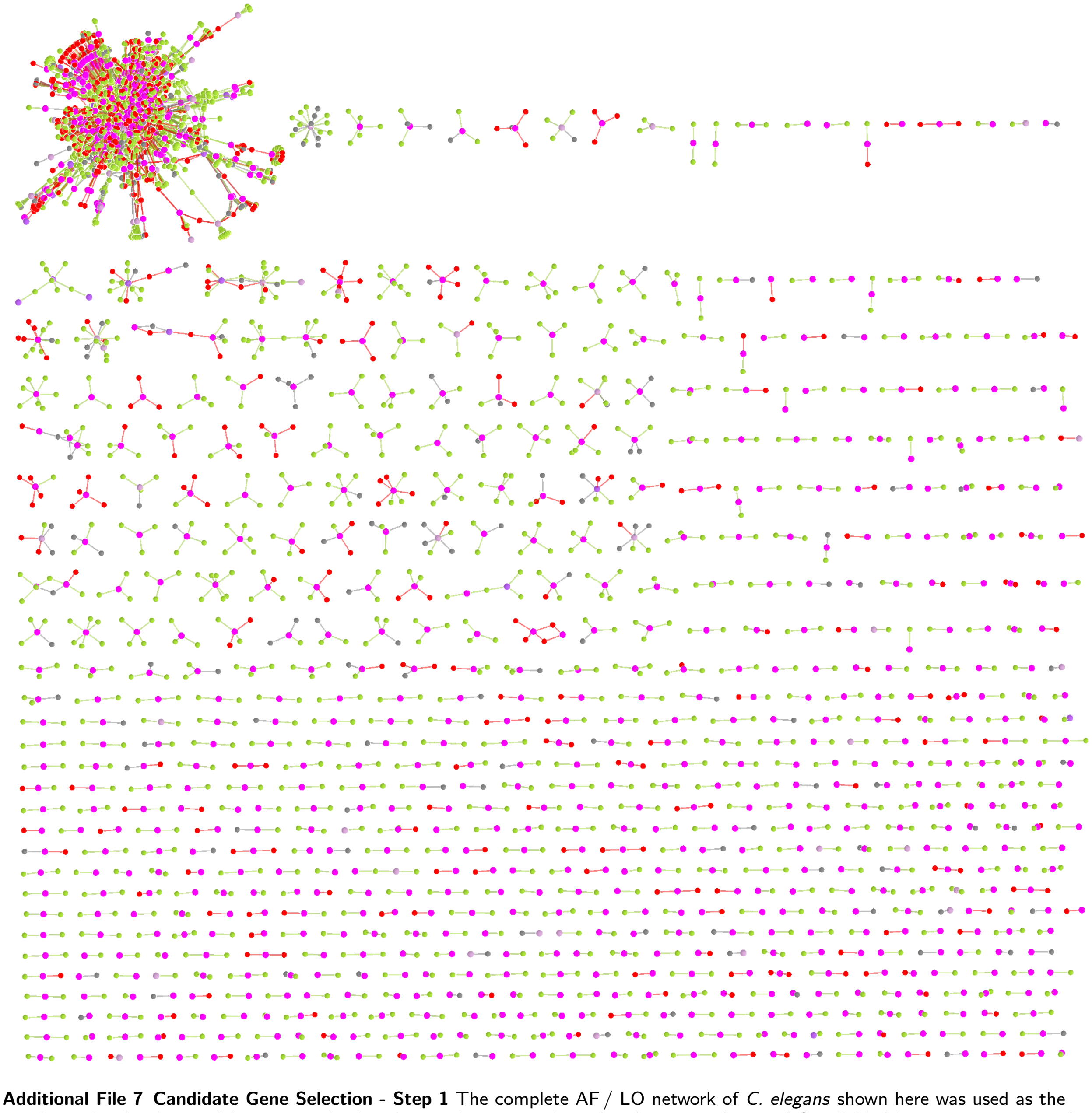

Additional File 7 Candidate Gene Selection - Step 1 The complete AF / LO network of *C. elegans* shown here was used as the starting point for the candidate gene selection. It contains 965 ageing-related genes and 4,265 LOs, divided into 676 unconnected subnetworks. The qualitative lifespan effect is encoded in the LO node and edge colour, according to the colour scheme below. **Network size:** 5,318 nodes, 5,874 edges; **Layout calculation:** FMMM algorithm with standard parameters; **Color scheme:** ● AF - gene, ● AF - compound, ● AF - other factor, ● LO - increased lifespan, ● LO - decreased lifespan, ● LO - unchanged lifespan

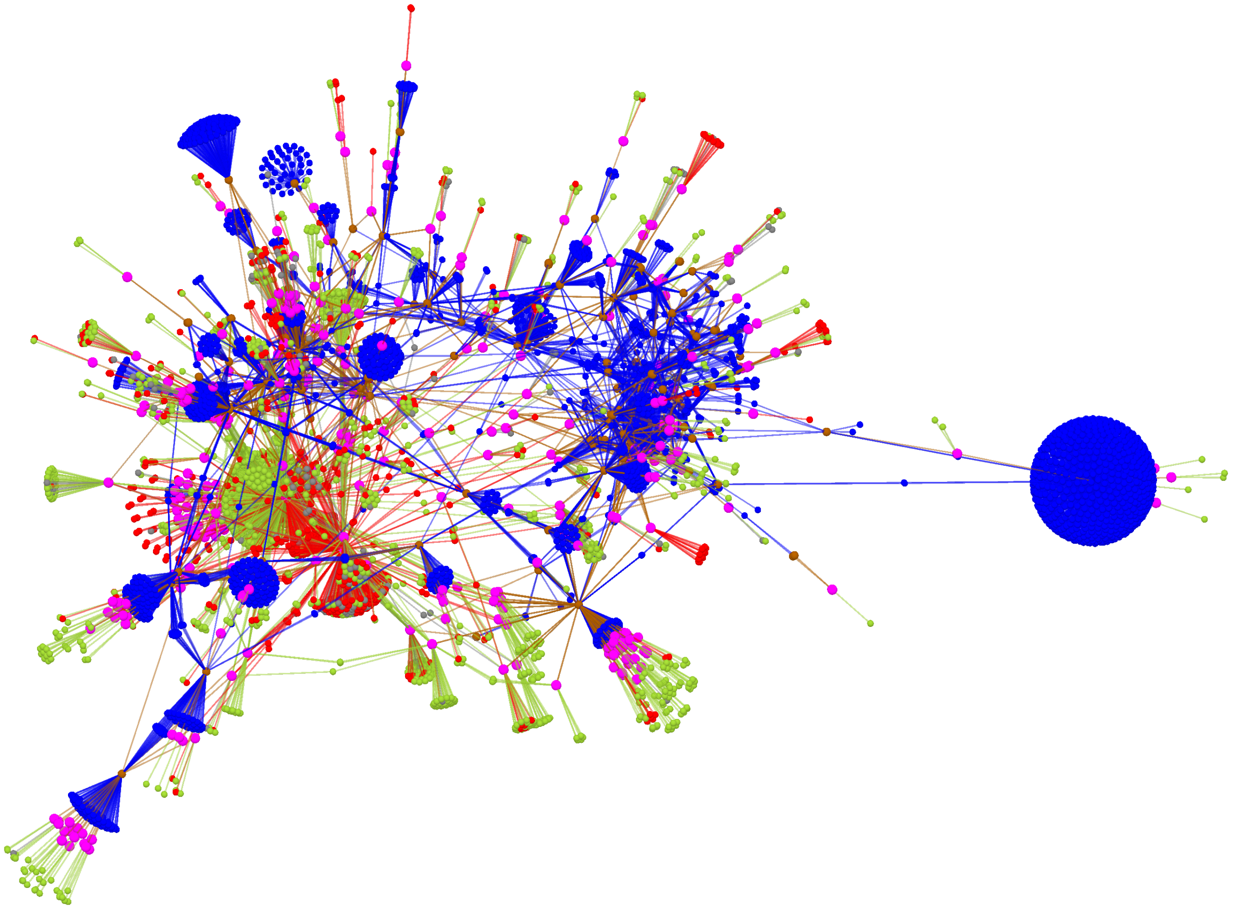

Additional File 8 Candidate Gene Selection - Step 2 For the second step, the KEGG pathway / NCBI Gene cross-linking information from the BioSystems database was used to expand the AF/LO network. Pathway nodes were added for all *Caenorhabditis elegans* specific pathways linked to AFs in AgeFactDB and gene nodes for all other genes connected to these pathway nodes. AFs not connected to any pathway node were removed, together with LOs only connected to them. Through the expansion of the network with genes linked to the same KEGG pathways as AFs, a large number of potential new ageing-related genes were added. The qualitative lifespan effect is encoded in the LO node and edge colour, according to the colour scheme below. **Network size:** 5,263 nodes, 8,150 edges; **Layout calculation:** FMMM algorithm with standard parameters; **Color scheme:** ● AF - gene, ● LO - increased lifespan, ● LO - decreased lifespan, ● LO - unchanged lifespan, ● KEGG pathway, ● gene (from pathway and not in AgeFactDB)

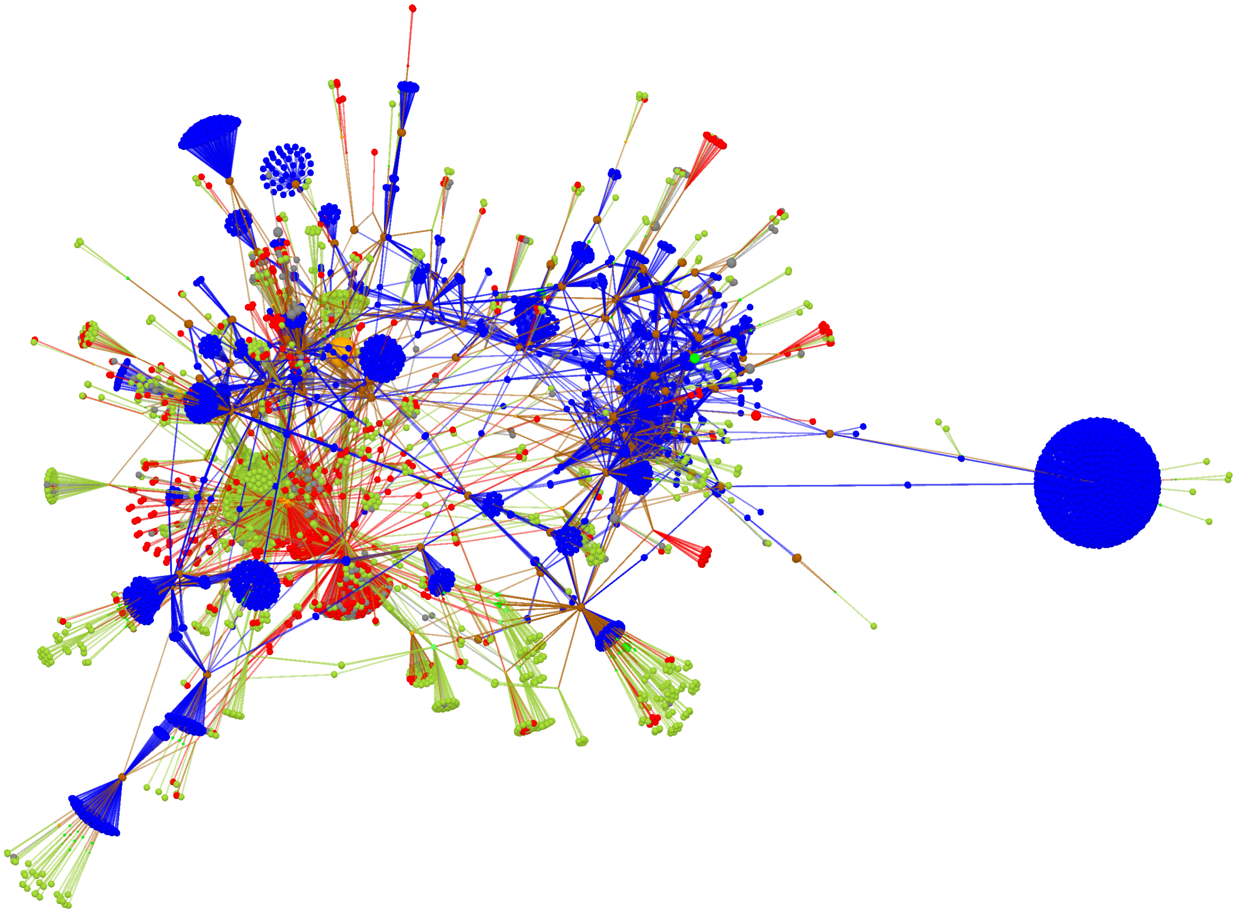

Additional File 9 Candidate Gene Selection - Step 3 In step 3, a summary of the LO information was transferred to the AF nodes, as described for the network in Figure 3D. In addition, the largest lifespan change observed for each AF was used to determine the size of each AF node. To facilitate the detection of the changes, the same layout was used as in step 2 (Additional File 8). The qualitative lifespan effect is encoded in the LO node and edge colour, according to the colour scheme below. Two colours are used for AFs and for LOs.
Network size: 5,263 nodes, 8,150 edges; **Layout calculation:** FMMM algorithm with standard parameters;
Color scheme: ● AF - LOs with increased lifespan (opaque: only, transparent: $\geq$80%), ● AF - LOs with decreased lifespan (opaque: only, transparent: $\geq$80%), ● AF - LOs with highly mixed lifespan changes (increased and decreased >20%), ● LO - increased lifespan (small), ● LO - decreased lifespan (small), ● LO - unchanged lifespan, ● KEGG pathway, ● gene (from pathway and not in AgeFactDB)

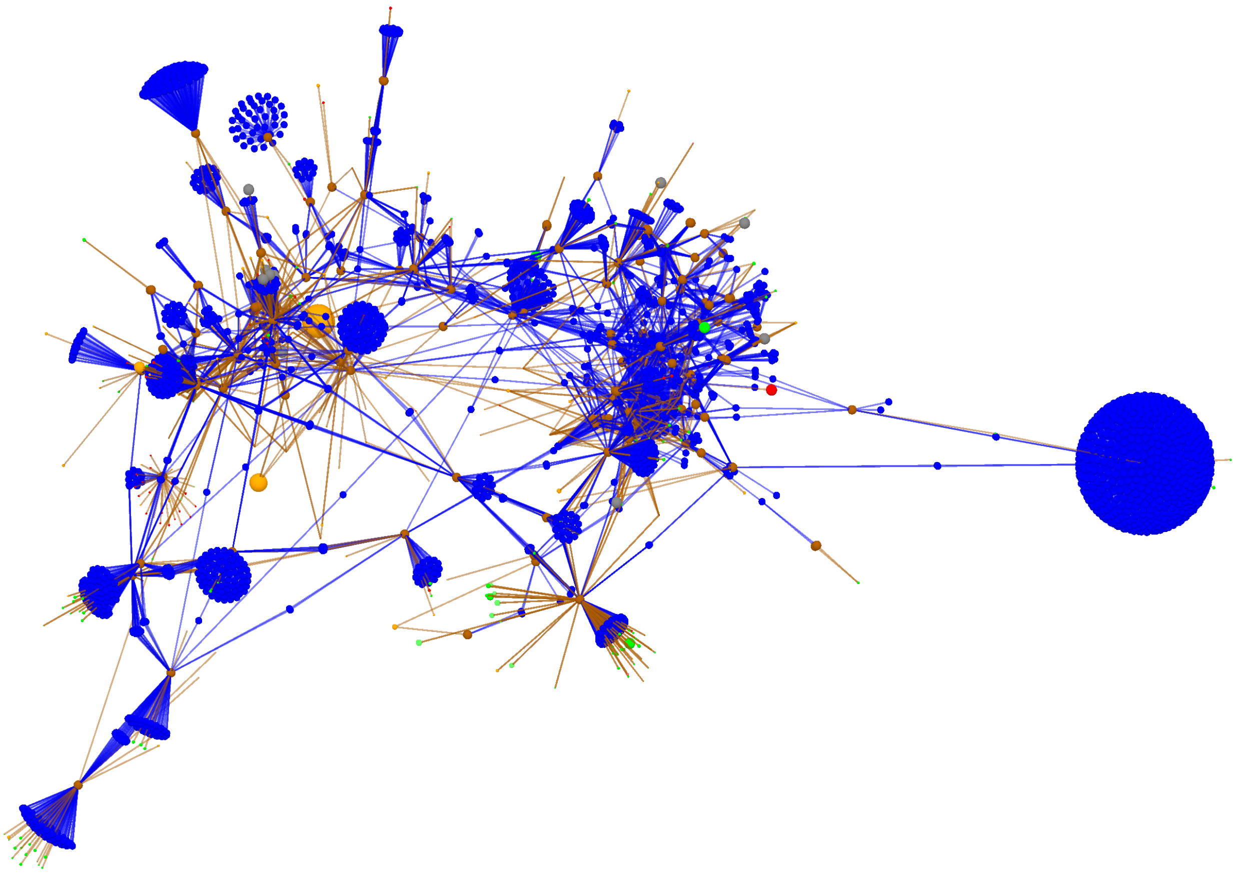

Additional File 10 Candidate Gene Selection - Step 4 In step 4, the complexity of the network was reduced by hiding the LO nodes, while retaining lifespan change information through the transfer done in step 3 (Additional File 9). To facilitate the detection of the changes, the same layout was used as in step 3. The summarised lifespan change information is encoded in the AF node size and node colour according to the colour scheme below.
Visible network size: 3,104 nodes, 5,522 edges; **Layout calculation:** FMMM algorithm with standard parameters;
Color scheme: ● AF - LOs with increased lifespan (opaque: only, transparent: ≥80%), ● AF - LOs with decreased lifespan (opaque: only, transparent: ≥80%), ● AF - LOs with highly mixed lifespan changes (increased and decreased >20%), ● KEGG pathway, ● gene (from pathway and not in AgeFactDB)

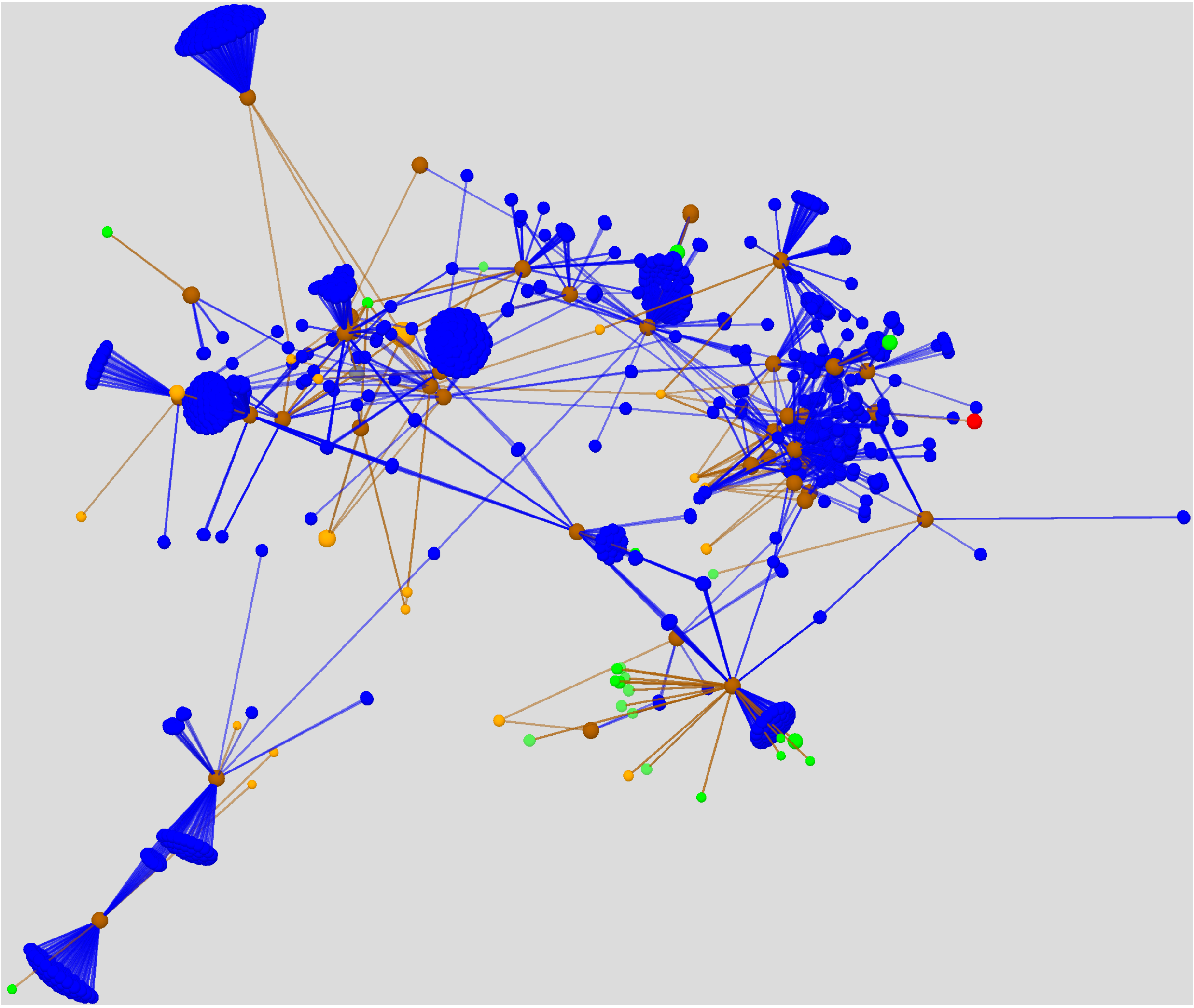

Additional File 11 Candidate Gene Selection - Step 5 In step 5, a first reduction of the number of candidate genes was done by hiding AF nodes with an assigned lifespan change of less than 100% and by hiding their connected nodes. This led to 786 candidate genes connected to 46 pathway nodes. To facilitate the detection of the changes, the same layout was used as in step 4 (Additional File 10). The summarised lifespan change information is encoded in the AF node size and node colour according to the colour scheme below.
Visible network size: 870 nodes, 894 edges; **Layout calculation:** FMMM algorithm with standard parameters;
Color scheme: AF - LOs with increased lifespan (opaque: only, transparent: ≥80%), AF - LOs with decreased lifespan (opaque: only, transparent: ≥80%), AF - LOs with highly mixed lifespan changes (increased and decreased >20%), KEGG pathway, gene (from pathway and not in AgeFactDB)

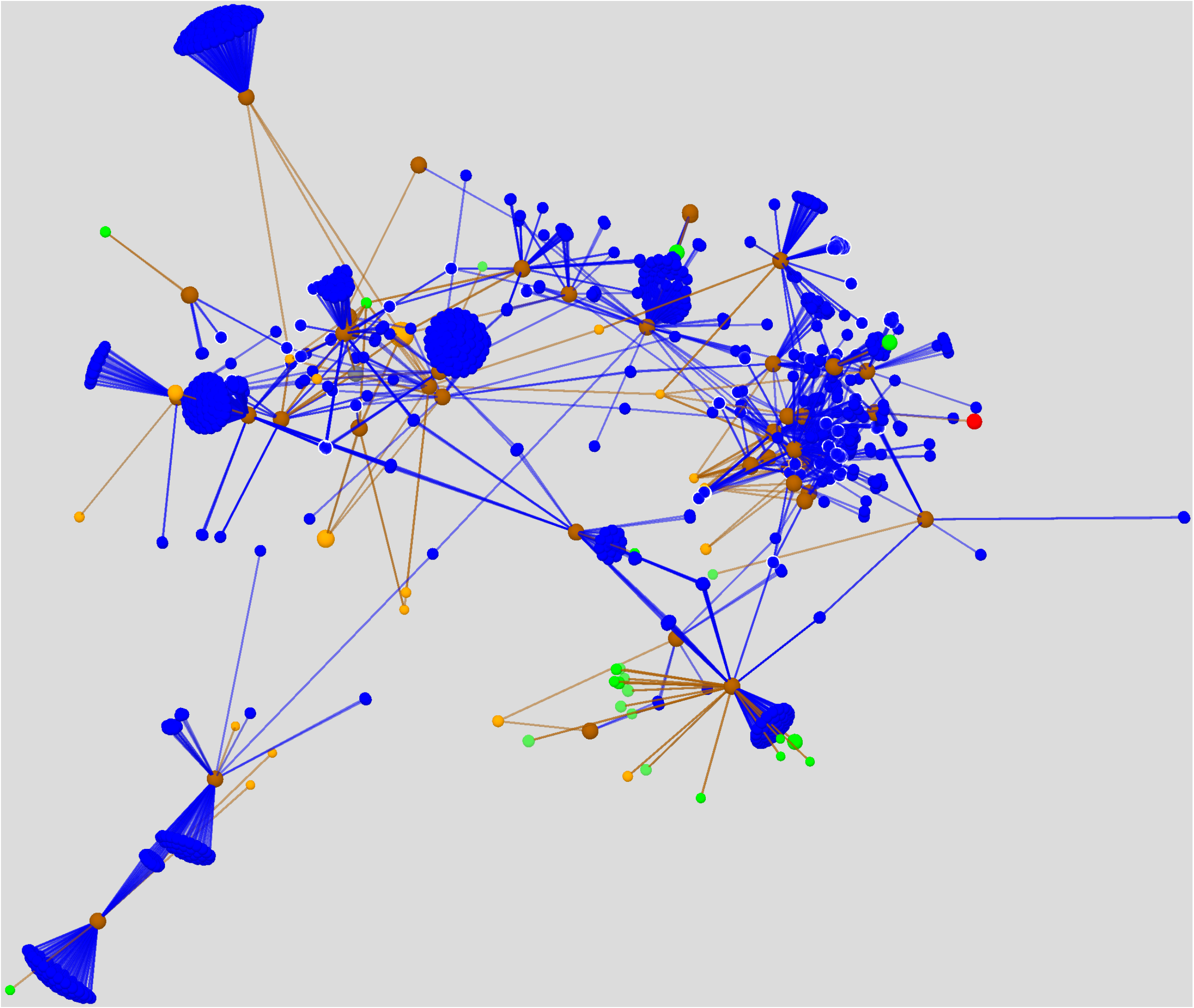

Additional File 12 Candidate Gene Selection - Step 6 In step 6, a second reduction of the number of candidate genes was done by selecting only genes connected to at least six pathway nodes. Selected genes are marked by a halo. To facilitate the detection of the changes, the same layout was used as in step 5 (Additional File 11). The summarised lifespan change information is encoded in the AF node size and node colour according to the colour scheme below.
Visible network size: 870 nodes, 1383 edges; **Layout calculation:** FMMM algorithm with standard parameters;
Color scheme: ● AF - LOs with increased lifespan (opaque: only, transparent: ≥80%), ● AF - LOs with decreased lifespan (opaque: only, transparent: ≥80%), ● AF - LOs with highly mixed lifespan changes (increased and decreased >20%), ● KEGG pathway, ● gene (from pathway and not in AgeFactDB), ● selected candidate gene

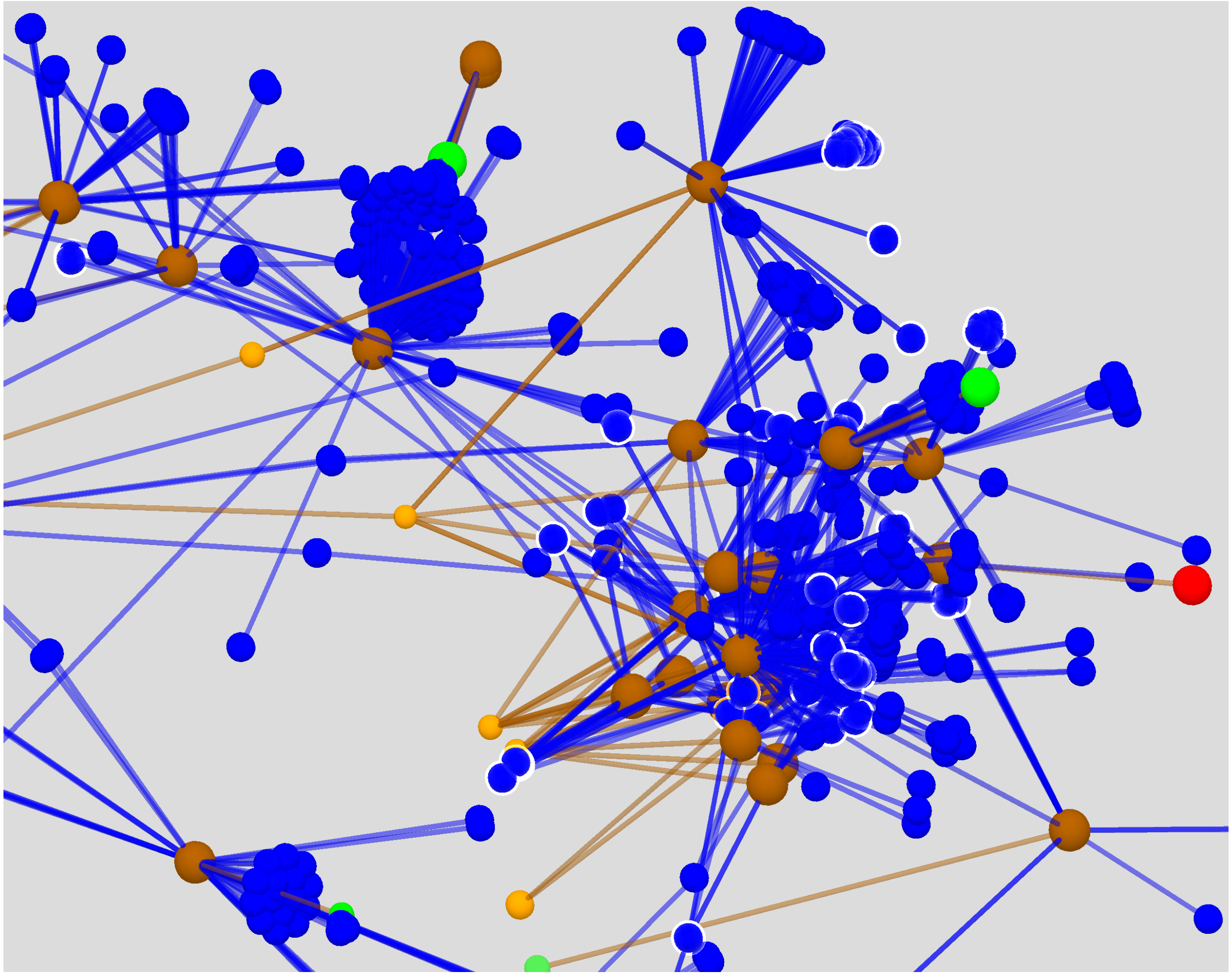

Additional File 13 Candidate Gene Selection - Step 7 In step 7, it was zoomed into the region of the network from step 6 (Additional File 12) with the largest number of selected candidate genes, marked by a halo, for a more detailed view. For all 95 selected candidate genes we did a literature search, summarised in Table 4. The summarised lifespan change information is encoded in the AF node size and node colour according to the colour scheme below.
Visible network size: 870 nodes, 1353 edges (partially clipped off); **Layout calculation:** FMMM algorithm with standard parameters; **Color scheme:** ● AF - LOs with increased lifespan (opaque: only, transparent: ≥80%), ● AF - LOs with decreased lifespan (opaque: only, transparent: ≥80%), ● AF - LOs with highly mixed lifespan changes (increased and decreased >20%), ● KEGG pathway, ● gene (from pathway and not in AgeFactDB), ● selected candidate gene

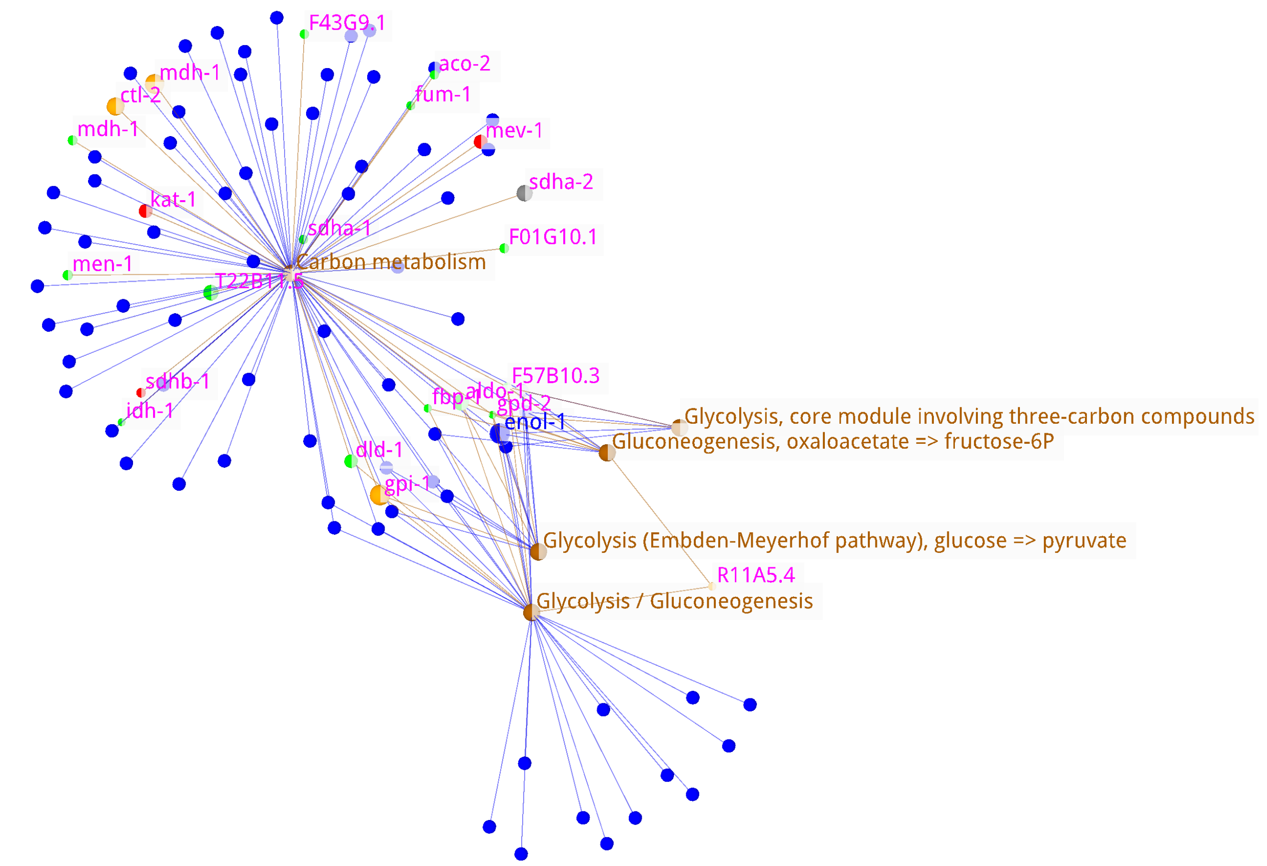

Additional File 14 Candidate Gene Selection - Step 8 In step 8, it was focused on the candidate gene *enol-1*, marked by a halo and by an increased node size. It was zoomed into the network from step 4 (Additional File 10), before the reduction of KEGG pathway and gene nodes. All nodes were hidden that are not connected to the seven pathway nodes which are connected to the gene *enol-1*. And the candidate gene node, the AF nodes, and the pathway nodes are labelled with their names, for a quick more detailed overview. The summarised lifespan change information is encoded in the AF node size and node colour according to the colour scheme below.
Visible network size: 162 nodes, 237 edges; **Layout calculation:** FMMM algorithm with standard parameters;
Color scheme: AF - only LOs with increased lifespan, AF - only LOs with decreased lifespan, AF - LOs with highly mixed lifespan changes (increased and decreased >20%), KEGG pathway, gene (from pathway and not in AgeFactDB)

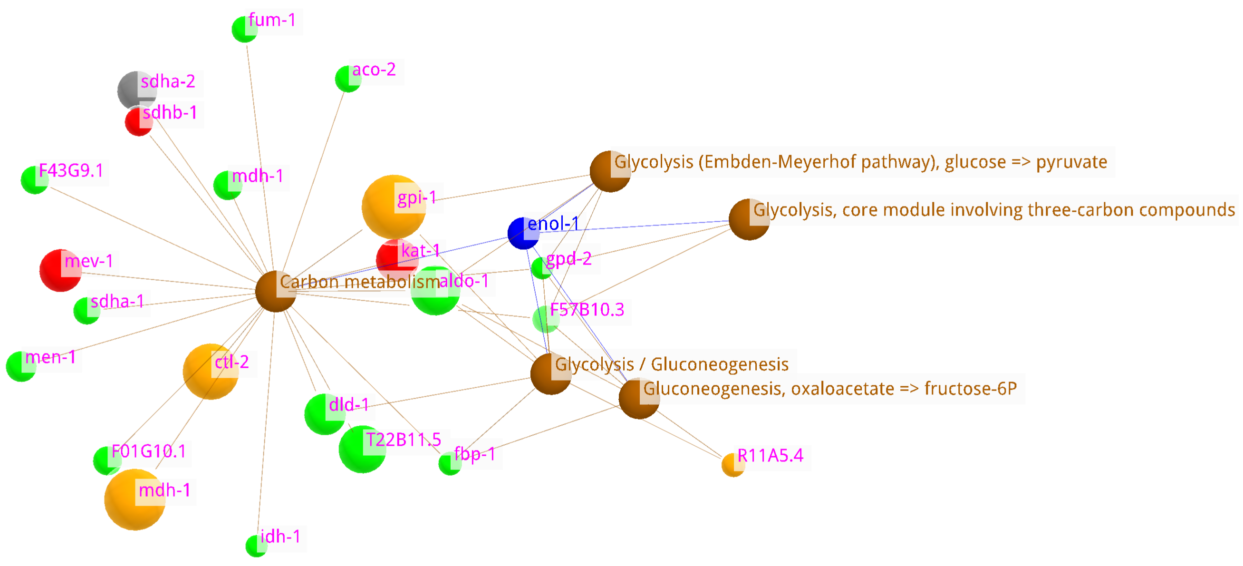

Additional File 15 Candidate Gene Selection - Step 9 In step 9, it was focused on the candidate gene *enol-1*, like in step 8 (Additional File 14). But in contrast to step 8, a new network was created with a new layout for an optimised view. And no other potential candidate genes were included, only the 7 pathway nodes connected to the gene *enol-1* and the AFs connected to them. Like before, information from the LOs was transferred to the AFs and visualised by node size and node colour according to the colour scheme below.
Network size: 40 nodes, 63 edges; **Layout calculation:** FR algorithm with standard parameters,
Color scheme: ● AF - only LOs with increased lifespan, ● AF - only LOs with decreased lifespan, ● AF - only LO with unchanged lifespan, ● AF - LOs with highly mixed lifespan changes (increased and decreased >20%), ● KEGG pathway, ● gene (from pathway and not in AgeFactDB)
